# Trait evolution during a rapid global weed invasion despite little genetic differentiation

**DOI:** 10.1111/eva.13548

**Published:** 2023-04-18

**Authors:** Ramona E. Irimia, Daniel Montesinos, Anurag Chaturvedi, Ian Sanders, José L. Hierro, Gastón Sotes, Lohengrin A. Cavieres, Özkan Eren, Christopher J. Lortie, Kristine French, Adrian Christopher Brennan

**Affiliations:** ^1^ Centre for Functional Ecology, Department of Life Sciences University of Coimbra Coimbra Portugal; ^2^ Plant Evolutionary Ecology, Institute of Evolution and Ecology University of Tübingen Tübingen Germany; ^3^ Australian Tropical Herbarium James Cook University Queensland Cairns Australia; ^4^ Department of Ecology and Evolution University of Lausanne Lausanne Switzerland; ^5^ Environmental Genomics Group, School of Biosciences University of Birmingham Birmingham UK; ^6^ Laboratorio de Ecología, Biogeografía y Evolución Vegetal (LEByEV), Instituto de Ciencias de la Tierra y Ambientales de La Pampa (INCITAP), Consejo Nacional de Investigaciones Científicas y Técnicas (CONICET) Universidad Nacional de La Pampa (UNLPam) Santa Rosa Argentina; ^7^ Departamento de Biología, Facultad de Ciencias Exactas y Naturales, UNLPam Santa Rosa Argentina; ^8^ Departamento de Botánica, Facultad de Ciencias Naturales y Oceanográficas Universidad de Concepción Concepción Chile; ^9^ Instituto de Ecología y Biodiversidad (IEB) Santiago Chile; ^10^ Aydın Adnan Menderes Üniversitesi, Biyoloji Bölümü, Fen‐Edebiyat Fakültesi Aydın Turkey; ^11^ Department of Biology York University Ontario Toronto Canada; ^12^ The National Center for Ecological Analysis and Synthesis (NCEAS), UCSB California USA; ^13^ School of Earth, Atmospheric and Life Sciences University of Wollongong New South Wales Wollongong Australia; ^14^ Department of Biosciences University of Durham Durham UK

**Keywords:** biogeography, divergent selection, invasive alien species, *P*_ST_–*F*_ST_ comparison, reproductive strategy, single‐nucleotide polymorphisms, yellow starthistle

## Abstract

Invasive species often possess a great capacity to adapt to novel environments in the form of spatial trait variation, as a result of varying selection regimes, genetic drift, or plasticity. We explored the geographic differentiation in several phenotypic traits related to plant growth, reproduction, and defense in the highly invasive *Centaurea solstitialis* by measuring neutral genetic differentiation (*F*_ST_), and comparing it with phenotypic differentiation (*P*_ST_), in a common garden experiment in individuals originating from regions representing the species distribution across five continents. Native plants were more fecund than non‐native plants, but the latter displayed considerably larger seed mass. We found indication of divergent selection for these two reproductive traits but little overall genetic differentiation between native and non‐native ranges. The native versus invasive *P*_ST_–*F*_ST_ comparisons demonstrated that, in several invasive regions, seed mass had increased proportionally more than the genetic differentiation. Traits displayed different associations with climate variables in different regions. Both capitula numbers and seed mass were associated with winter temperature and precipitation and summer aridity in some regions. Overall, our study suggests that rapid evolution has accompanied invasive success of *C*. *solstitialis* and provides new insights into traits and their genetic bases that can contribute to fitness advantages in non‐native populations.

## 1 INTRODUCTION

Populations of invasive alien species occur in a range of environments with particular abiotic conditions and biotic selection pressures, which often result in rapid phenotypic changes in the new habitat (Blanquart et al., 2013; Bossdorf et al., 2005; Montesinos, 2021). Many factors, such as gene flow, genetic drift, mutations, and standing genetic variation, can influence the extent and rate of population differentiation and create a mosaic of geographically independent populations in terms of evolutionary potential (Colautti et al., 2009; Lai et al., 2019; Matesanz et al., 2012; North et al., 2011). The success of an invader is determined by its ability to respond to the conditions it encounters and its rate of adaptation, which is influenced by the interplay between demographic and genetic processes and dispersal abilities (García‐Ramos & Rodríguez, 2002). Evidence of rapid evolution during species colonization appears to be a common feature of biological invasions and can increase invader fitness and its impact on the community (Bossdorf et al., 2005; Colautti & Lau, 2015; Prentis et al., 2008). Consequently, biological invasions present unique opportunities to study contemporary evolution and species range limits (Keller & Taylor, 2008; Lee, 2002), with a large body of research devoted to understanding the factors that contribute to invasion success (Enders et al., 2020). Integrating knowledge from invasion biology about species evolutionary responses can be highly informative in terms of biodiversity conservation and predicting species range expansion in the context of climate change (Bock et al., 2015).

Identifying and understanding the processes and mechanisms behind invasive species colonization, establishment, and range expansion, as well as the role played by genetic diversity in invasion success and the traits associated with invasiveness, and how they evolve, remain central to the field of invasion genetics (Bock et al., 2015). During the past 15 years, this has benefited from the rapid progress in genomic approaches that allow us to capture patterns of genome‐wide variation and add a much finer resolution into several aspects of the invader success including the colonization history, population demography, and evolution of invasiveness (Matheson & McGaughran, 2022; McGaughran et al., 2021; North et al., 2021). Different species traits have been identified as important determinants of invasive success including a fast growth rate, short life cycles, high reproductive output, good dispersal ability, and a high resource acquisition capacity (Hodgins et al., 2018). However, given the complexity and dynamics of the invasion process, different sets of traits appear to be important under different contexts and at different stages of invasion (Hodgins et al., 2018; van Kleunen et al., 2015). For species that have recently become invasive, comparison of genotypes and phenotypes in their novel environments relative to that of their native environments (“home *vs*. away”; Pigliucci, 2001; Hierro et al., 2005) can be highly informative about the phenotypic traits that vary between invasive and non‐invasive genotypes, the role of selection and the genetic architecture of invasiveness (Blanquart et al., 2013; Bock et al., 2015; Keller & Taylor, 2008). For example, Turner et al. (2021) used genotyping by sequencing (GBS) and conducted a genome‐wide association study (GWAS) on native and invasive populations of *Centaurea diffusa* Lam. grown under common garden conditions for two generations. They found that invasive plants have significantly larger leaves compared with native plants, a trait that is associated with increased biomass and fitness, and they also identified several potential candidate genes for local adaptation (Turner et al., 2021). Recent studies of native and introduced populations of *Mimulus guttatus* Fisch. ex DC. generated genome‐wide SNPs markers and conducted demographic analyses to reconstruct the species global invasion and understand how the species may evolve toward increased invasiveness (Puzey & Vallejo‐Marín, 2014; Vallejo‐Marín et al., 2021). Another study by Hodgins et al. (2012) used a microarray experiment to examine differences in gene expression patterns between native and introduced populations of *Ambrosia artemisiifolia* L. under light and nutrient stress treatments but found no consistent pattern of up‐ or down‐regulation of these genes between ranges. Although during the last decade we have gained important insights into the molecular mechanisms of species adaptation, our understanding of how the genotype translates into phenotype in response to different environments remains limited (Neinavaie et al., 2021).

A powerful approach to study adaptive evolutionary changes in populations of invasive species is to compare the genetically determined quantitative trait differentiation (termed *Q*_ST,_ Spitze, 1993; Leinonen et al., 2013) with neutral genetic differentiation (*F*_ST_, Wright, 1951). However, estimation of *Q*_ST_ requires prior knowledge about relatedness between sample individuals, which for some species might not be feasible. An alternative to *Q*_ST_ is *P*_ST_ in these cases. The phenotypic differentiation index (*P*_ST_) uses purely phenotypic data to compare the combined influence of genetic adaptation, phenotypic plasticity, and genetic drift as causes of population differentiation (Leinonen et al., 2006, 2013). Under the neutral expectation, *P*_ST_ equals *F*_ST_, with any significant deviation indicative of an additional influence of selection on *P*_ST_ (i.e. a *P*_ST_ < *F*_ST_ is evidence of stabilizing selection while a *P*_ST_ > *F*_ST_ indicates a history of adaptive divergence, Leinonen et al. (2006).

*Centaurea solstitialis* L. (yellow starthistle, Asteraceae) is an outcrossing annual diploid herb (Irimia et al., 2017) with a genome size of 1C = 851 Mbp (Bancheva & Greilhuber, 2006), native to the Mediterranean region. Seeds of *C*. *solstitialis* were introduced across the world during the past 200–250 years as a contaminant of alfalfa seed and established invasive populations in South America, North America, South Africa, and Australasia (DiTomaso et al., 2006). Previous work using population genomic analyses across a broad range of *C*. *solstitialis* in Eurasia, North America, and South America has showed that native populations are genetically and geographically structured into four groups including an Asian group (Turkey, Armenia, and Uzbekistan), an Eastern European group, a southern Greece group, and a Western European group (Barker et al., 2017). The same study found evidence that populations in Western Europe are derived from an ancient admixture event among populations from Eastern Europe and Asia and that they served as a bridgehead source for introductions into the Americas (Barker et al., 2017). Moreover, the study by Barker et al. (2017) identified similar levels of genetic diversity between native and introduced ranges and overall low genetic structure in the invaded range as well as evidence of multiple introductions from different sources in some areas of North America. Several phenotypic studies in this species demonstrated a higher performance of invasive genotypes compared to native genotypes including an increase in plant size (Barker et al., 2017; Dlugosch et al., 2015; Eriksen et al., 2012), increased seed size (Eren & Hierro, 2021; Hierro et al., 2013, 2020), and increased biomass production (Montesinos & Callaway, 2018a, 2018b; Widmer et al., 2007), faster growth rates (Graebner et al., 2012; Montesinos & Callaway, 2018a, 2018b), earlier flowering time (Dlugosch et al., 2015; Eriksen et al., 2012), increased leaf chemical defenses (Sotes et al., 2015), and higher reproductive output (Dlugosch et al., 2015), which suggests that rapid local adaptation has occurred in the introduced range. Still, there are few genetic studies in this species that have aimed to gain insights into evolutionary forces and identify potential adaptive traits contributing to fitness advantages and invasion across the global invasive range. Thus, there is great potential to combine the power of genomic tools with ecological studies to collate such information for this invader.

In this study, we used a combination of approaches to search for genetic signals of divergent selection in *C*. *solstitialis*. First, we assessed trait differences among regions and between the native and non‐native ranges to gain insights into their evolutionary divergence (*P*_ST_–*F*_ST_ comparison) by growing plant individuals under a common garden environment. We then tested for associations between the phenotypic traits showing differentiation and the environmental variables at the sites of origin. In addition, we included samples from Australia – a region not considered in previous genetic and evolutionary studies of yellow starthistle, to have additional introduced populations with which to test for selection and trait differentiation.

## 2 MATERIALS AND METHODS

### 2.1 Study species and seed collection

**FIGURE 1:**
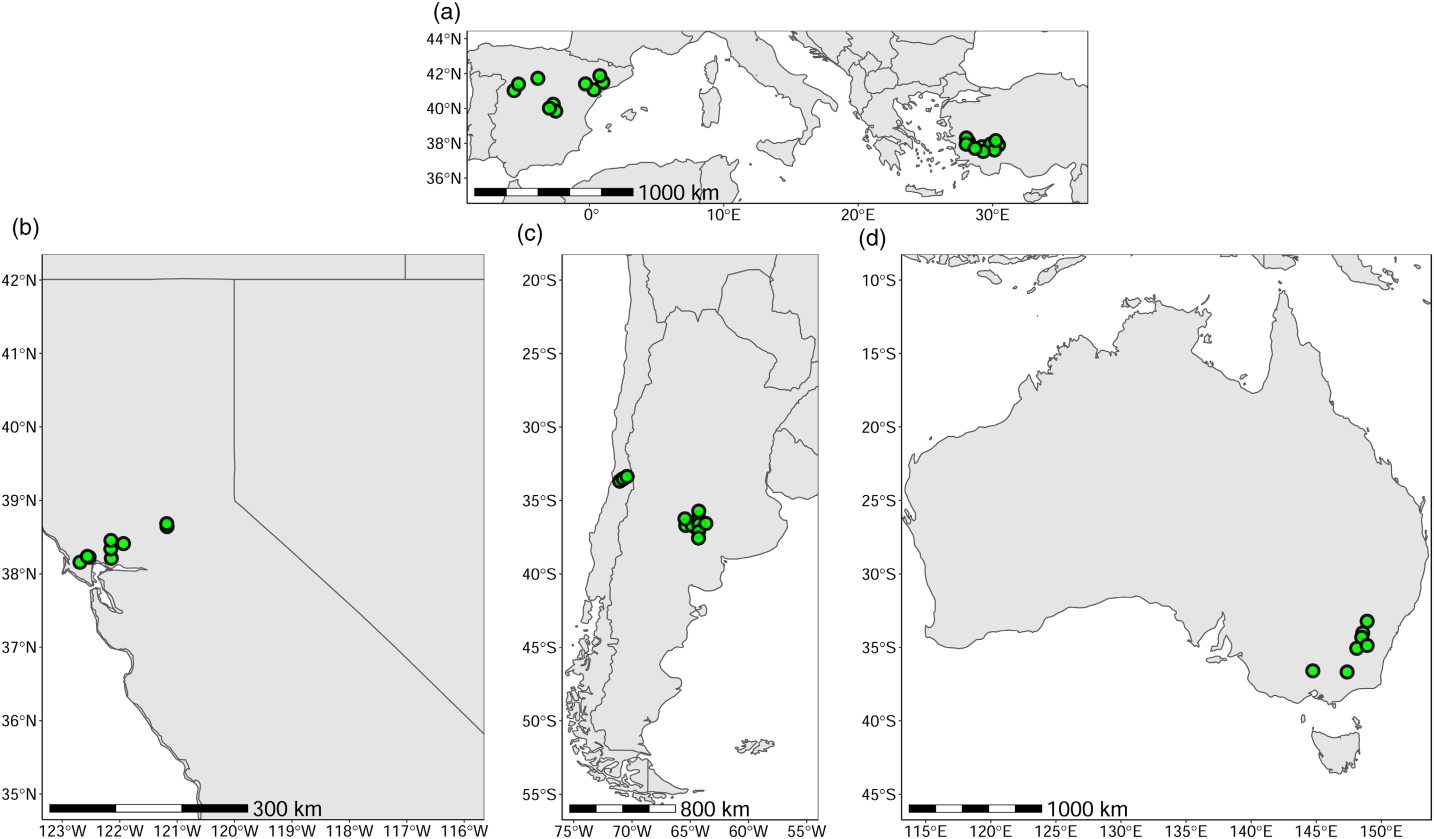
*Centaurea solstitialis* sampling sites in (a) Turkey and Spain (native), and non‐native regions of (b) California coast, (c) central Argentina and Chile, and (d) SE Australia. Each dot corresponds to a population.

A total of 50 *C*. *solstitialis* populations were sampled in the wild between 2009 and 2014 spanning sites in the species ancestral range in Asia (Anatolia, Turkey) and expanded range in Western Europe (Spain), as well as introductions in South America (central Argentina and central Chile), North America (California coast), and south eastern Australia. Both the ancestral and expanded ranges are considered to be part of *C*. *solstitialis* native range (Hierro et al., 2020). At least 30 individuals were sampled within each population, and population sampling aimed to include a broad geographic and environmental range for each country (hereafter referred to as regions). Ten populations were from Turkey, 10 from Spain, 10 from Argentina, four from Chile, nine from California (USA), and seven from Australia (see Table S1 and Figure 1 for information on sampling sites).

### 2.2 Common garden experiment

Seeds were germinated in a glasshouse at the Botanical Garden of the University of Coimbra in 50 cell plug trays containing commercial soil (Substratos Profissionais Leal and Soares S.A., Portugal) and seedlings were transplanted into 2 L square plastic pots (one plant per pot) filled with the same type of potting soil about 3 weeks after germination. Plants were kept in an insect excluded glasshouse where they experienced a Mediterranean climate (sunny and hot summer which is typical for Coimbra) and were grown to maturity and senescence (March–October 2017, which overlaps with the plant life cycle in the wild). We recorded six phenotypic traits on each individual that reached reproductive stage and survived through senescence (*N* = 370) including (1) days to bolting; (2) days to first flower; (3) length of the largest spine; (4) final plant height; (5) capitula number; and (6) seed mass. These traits relate to plant growth, reproductive output and herbivore defense (flower spinescence) and were shown to be important to *C*. *solstitialis* invasive success (Agrawal et al., 2000; Barker et al., 2017; Dlugosch et al., 2015; Hierro et al., 2020). To obtain an F1 generation of seeds for seed mass weighing, we performed experimental crosses between individuals within each of the 50 populations included in this study (between different individuals belonging to the same population i.e., intra‐population, see methodology in Irimia et al., 2021) and scored the mass of the seeds with pappus (fibrous outgrowths) for the total number of seeds produced. Final plant height (stem base to the highest point) and spine length (base to tip) were measured to the nearest millimeter using a flexible ruler, at the end of the experiment. A plant was considered to have initiated bolting when a flowering stem of ~5 cm tall started to extend from the basal rosette. At the end of the reproduction period, we counted all the capitula and lateral floral buds on each individual. Once the fruit ripened, we dissected the capitulum and counted the total number of ovules and viable seeds. Seeds obtained from the controlled crosses were stored in paper bags at room temperature for 6 months and then weighed on a Kern ALJ analytical balance (Balingen, Germany) to the nearest milligram.

### 2.3 Environment characterization

We obtained climate data for the period 1970–2000 for all 50 maternal field sites from WorldClim 2 (Fick & Hijmans, 2017, see www.worldclim.com/version2), at a spatial resolution of 2.5 arc‐minutes. The data consisted of 19 bioclimatic variables related to temperature (bio1‐–bio11) and precipitation (bio12–bio19) for different periods of a year. To quantify climatic differences among regions, we used radar plots and conducted a standardized and centered PCA (principal component analysis) using the (*prcomp*) function in the package factoextra (Kassambara & Mundt, 2016). We generated a PCA biplot and radar plots to visualize population distribution in the climatic space.

### 2.4 ddRADSeq library preparation

We selected 194 individuals sampled across the six regions for double digest DNA (ddRADSeq), a reduced representation sequencing and genotyping method that has previously been applied with success to this species (Barker et al., 2017). Genomic DNA was isolated from silica gel dried leaves sampled from 8‐week‐old individual plants grown in the glasshouse, using the CTAB protocol (Doyle & Doyle, 1987). Leaf tissue was ground to a fine powder with the Tissue Lyser II (Qiagen) for 2 min at 25 Hz. Following extraction, DNA quality and quantity was assessed on a Nanodrop 2.0 spectrophotometer (Thermo Fisher) and a Qubit 2.0 fluorometer (Invitrogen Life Technologies) using the QuantiFlour dsDNA sample kit (Promega). A starting quantity of 500 ng of purified DNA from each individual was digested with the restriction enzymes, *Pst1* (recognized sequence and cut site: *CTGCA^G*), and *Mse1* (*T*^*TAA*), followed by the ligation of unique paired combinations of individual P1 and P2 barcoded adapters (Table S2) following the protocol by Barker et al., 2017. Equal amounts of barcoded DNA from each sample were pooled in a single library that was purified using SpeedBead Magnetic Carboxylate modified particles (GE Healthcare UK) and size‐selected for DNA fragments between 300 and 550 bp on a Pippin Prep automated size selection system (Sage Science) using a 2% agarose gel cassette (dye free 100–600 bp, DNA size range collection with external marker L; Sage Science). The size‐selected DNA library was amplified by 11 PCR cycles using Phusion High‐Fidelity DNA polymerase (New England BioLabs) on a Prime Thermal Cycler (Midwest Scientific), to increase the concentration of properly ligated DNA fragments, and the resulting product was purified with SpeedBead modified particles and then eluted in 10 μM TrisHCl‐EDTA solution. Library fragment size distribution was visualized on an Agilent 2200 TapeStation System by testing 1 μl of purified DNA library using the D1000 high sensitivity ScreenTape and D1000 Sample buffer (Agilent Technologies). Quantitative PCR (qPCR) was done using the NEBNext Library quantification kit for Illumina (New England Biolabs) to measure the concentration of the pooled library on a BioRad CFX connect real time system (BioRad). Libraries were sequenced on five separate lanes of an Illumina HiSeq 2500 (Illumina, San Diego, CA, USA) at the University of Durham Genomics Sequencing and Analysis Facility, UK, to generate 125 bp paired‐end reads. In total, we obtained ~1.26 billion pair‐end Illumina reads across 194 individuals. Accessions and run information are available in NCBI under BioProject ID: PRJNA950038.

### 2.5 Data processing

#### 2.5.1 Morphological traits, phenotypic and genomic divergence indices

All analyses were conducted in R v3.5.2 (R Development Core Team, 2014). Phenotypic data were checked for heteroscedasticity and normality by Levene's and Shapiro–Wilk tests (Levene, 1960; Shapiro & Wilk, 1965). Trait associations were tested with Pearson's correlation tests. To test the differentiation of phenotypic traits among native (Turkey, Spain) and non‐native (Argentina, Australia, California, Chile) regions, we generated generalized linear mixed‐effects models by using the glmer function in the *lme4* package (Bates et al., 2015). We used region as a fixed factor and population as a random factor and germination time as a covariant to account for potential growth differences between different plants. If the models indicated significant differences among regions, we applied Tukey HSD post hoc tests with *p*‐values < 0.05 to infer which pairs of regions differed. To test for differences in phenotypic traits between native vs. non‐native ranges, we generated generalized linear mixed‐effects models with range as a fixed factor and population nested by region as a random factor. We used the dropterm function to obtain differences in AIC values across models. We performed a principal component analysis (standardized and centered PCA), to visualize trait differences between the native and non‐native ranges of *C*. *solstitialis* by employing the prcomp function to compute principal component scores and plotted the PCA with the fviz_pca function in the *factoextra* package v1.0.3 (Kassambara & Mundt, 2016). We used the *psych* and *stats* packages in R (Revelle, 2018) to test for trait correlations, generate pairwise scatter plots, and compute Pearson correlation coefficients.

To compare the level of phenotypic and genetic divergence between native and non‐native populations, we calculated *P*_ST_, an index that assesses differentiation among populations in quantitative traits. *P*_ST_ for each trait and each paired region combination as well as between sets of regions combined into native and non‐native ranges was calculated using a Bayesian approach following Leinonen et al. (2006). We fitted a linear model with population and region as a random effect and the trait of interest as the response variable using a Gibbs sampler implemented in the software WinBUGS 1.4.3 (Lunn et al., 2000) and specifying the corresponding mean value of *F*_ST_ (for *F*_ST_ calculation see the paragraph below on neutral population genetic structure). We assumed trait heritability to be 1 as is standard in these analyses (Leinonen et al., 2006). Posterior distributions were obtained by running five independent chains (50,000 iterations) after a burn‐in of 1000 iterations. Bayesian 97.5% credibility intervals were estimated for both *P*_ST_ and *P*_ST_–*F*_ST_ difference. If the credibility interval of the *P*_ST_–*F*_ST_ difference was higher than zero, we regarded it as an indication that the expression of the trait tested is putatively under selection. Alternatively, when the *P*_ST_–*F*_ST_ credibility interval overlaps zero, we interpreted that the observed degree of differentiation at quantitative traits could be the outcome of genetic drift (Leinonen et al., 2006, 2013).

#### 2.5.2 Phenotypic and environmental associations

To overcome collinearity, we used principal component analysis (PCA) to reduce the 19 bioclimatic variables to two or three main principal components that captured at least 85% of the total variance. We then used these principal components to build generalized linear mixed models to quantify the relationship between the phenotypic traits and the environmental characteristics of the maternal field site, at the regional level (i.e. within each region). We tested the effects of the fixed variables using the dropterm function and Chisq test.

#### 2.5.3 De novo SNP discovery

Sequencing data was demultiplexed or assigned to each sample individual according to their unique paired barcode sequences, and reads were quality filtered using Stacks 2.2 (Catchen et al., 2011, 2013) to remove reads containing adapter sequence and low‐quality base scores (a phred score below 10). The average number of reads per individual after demultiplexing and filtering was 6.5 M (range 300 K–44 M). All reads were end trimmed to 115 bp (combined paired read size 230 bp) to ensure that they were of the same length before running it through the denovo_map pipeline. We used only the complete paired reads in the mate files 1.fq and 2.fq to perform the alignment in denovo_map.pl with the following set of parameters: a minimum coverage depth of five to create a stack (*−m* = 5), a maximum mismatch distance of two nucleotides between loci when processing a single individual (*−M* = 2), a maximum of two stacks at a single locus (*−X* = 2), and a mismatch distance of two nucleotides between loci (*−n* = 2), to account for the possibility of fixed differences at loci in individuals when creating the catalog of loci, according to Barker et al. (2017). We also tested a higher stack depth (*−m* = 10), but this did not significantly change the results. The *C*. *solstitialis* individuals were grouped into six geographic regions by supplying the population map into the denovo pipeline. We obtained 526,533 variant sites (unfiltered) across 194 individuals. Fifty individuals had sequenced poorly with less than 500,000 reads each, so we decided to remove them and keep 144 individuals (24 per region × 6 regions) for all the subsequent analysis. VCFtools were used to filter the genotype data for the highest quality genotype calls by filtering out indels, including only bi‐allelic sites (–min‐alleles 2 –max‐alleles 2), including only a minimum genotyping proportion per population of 0.9, and retaining only sites with a minor allele frequency greater than 0.05 in the whole dataset. These stringent filtering steps retained high‐quality genotypes for 2138 variant sites (SNPs) across 144 individuals.

#### 2.5.4 Genomic signatures of selection

We conducted an outlier analysis to detect loci that were particularly differentiated and potentially linked to loci under selection. We used three methods to identify a consensus list of loci and minimize false positives that each individual method might find. The first software, bayescan, uses a Bayesian likelihood method that assumes a Dirichlet distribution of allele frequencies between populations and a reversible‐jump MCMC algorithm to calculate a posterior probability that each locus is under selection (Foll & Gaggiotti, 2008). We conducted 10 pilot runs each consisting of 5000 iterations, with a burn‐in of 50,000 iterations, a thinning interval of 10 and prior odds for neutral model set to 10, for a total of 3 replicates runs. The second software, OutFlank calculates a normal distribution of FST values from all SNPs and detects outliers using left and right‐tail trim fractions. This distribution is then used to assign *q*‐values to each locus to detect outliers that may be due to spatially heterogeneous selection (Whitlock & Lotterhos, 2015). Finally, the last software, PCAdapt is based on principal component analysis to detect outliers where each SNP is regressed against each principal component, with outliers extracted using z‐scores (Privé et al., 2020). Combining the results from each method, we compiled a list of putative outlier SNPs.

#### 2.5.5 Population neutral genetic structure

Genetic diversity of each region was calculated at 1975 neutral SNPs (see previous section that identified putatively outlier SNPs that were excluded from this analysis) as *H*_o_ (observed heterozygosity), *H*_e_ (expected heterozygosity)_,_ *AR* (allele richness), and *F*_IS_ (inbreeding coefficient) using the function divBasic in the R package diveRsity (Keenan et al., 2013). We calculated pairwise region differentiation and its 95% CI between native and non‐native ranges using the standardized allelic variance *F*_ST_ with the diveRsity package (Keenan et al., 2013). We estimated effective population size (Ne) using NeEstimator V2.1 (Do et al., 2014) and the linkage disequilibrium method assuming random mating (Sun & Ritland, 1998). We assessed neutral population genetic structure at 1975 SNPs in STRUCTURE 2.3.4 (Pritchard et al., 2000) by implementing a model of correlated allele frequencies (Falush et al., 2003) and admixture, and applying the default setting for all other parameters. Ten independent runs for all values of K (number of genetic clusters) between 1 and 8 were run using an MCMC length of 1,000,000 generations following a burn‐in of 100,000 generations in accordance with recommended practice (Gilbert et al., 2012). STRUCTURE HARVESTER (Earl & von Holdt, 2012) program was used to carry out downstream processing of STRUCTURE results to calculate Evanno's Δ*k* value to determine the optimal value of K (Evanno et al., 2005) and prepare an input file for CLUMPAK (Kopelman et al., 2015) to generate bar graphs of population structure. Population structure at neutral SNPs was also visualized using a discriminant analysis of principal components (DAPC) (Jombart et al., 2010) as implemented in the R packages, adegenet 2.1.1 (Jombart, 2008) and *ade4* (Dray & Dufour, 2007).

## 3 RESULTS

### 3.1 Phenotypic trait differentiation

**FIGURE 2:**
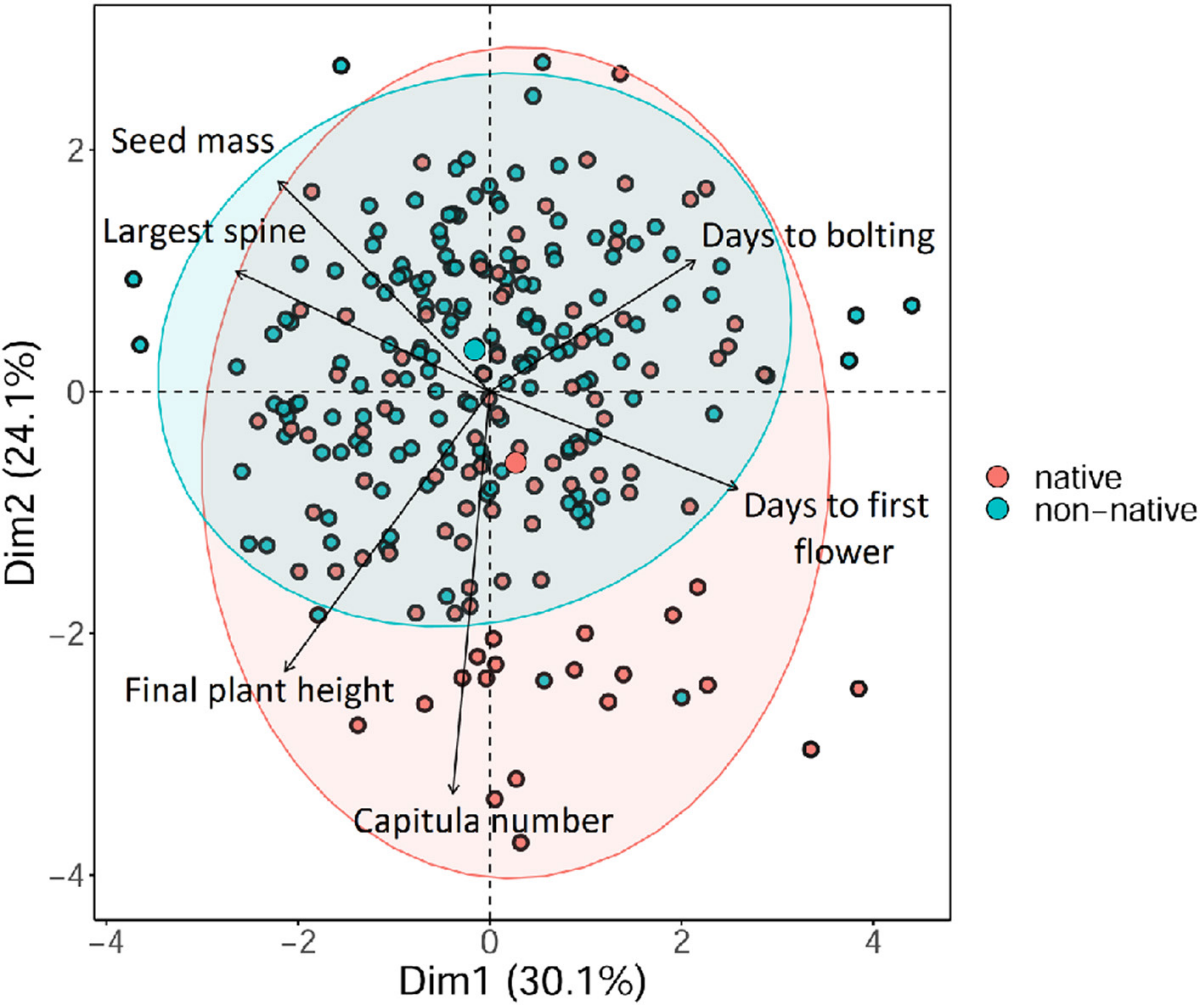
Principal component analysis (PCA) on six phenotypic traits – comparison between native and non‐native ranges. PCA1 and PCA2 together explained 54.2% of the inertia variance in the two axes. The first PC axis was negatively associated with the length of the largest spine and positively associated with the days to first flower while the second PC axis was negatively associated with the number of capitula. The larger symbol of the two groups represents the centroid (i.e., the average coordinates of samples in that group).

We detected differences between the native and non‐native ranges, but only for two traits namely capitula number (*χ* ^2^_(1)_ = 9.84, *p* = 0.001) and seed mass (*χ* ^2^_(1)_ = 4.31, *p* = 0.03). Native plants produced 54% more capitula compared with their non‐native counterparts, whereas introduced individuals produced seeds that were on average 34% heavier than those of native individuals (Figure 2). Five traits out of six showed significant differences among regions: (1) days to bolting: (*χ* ^2^_(1)_ = 11.52, *p* = 0.04); (2) days to first flower: (*χ* ^2^_(1)_ = 13.82, *p* = 0.01); (3) number of capitula: (*χ* ^2^_(1)_ = 27.87, *p* < 0.001); (4) length of largest spine: (*χ* ^2^_(1)_ = 11.17, *p* = 0.04) and (5) seed mass: (*χ* ^2^_(1)_ = 36.27, *p* < 0.001). Plants from California initiated bolting 7.7 days earlier than plants from Spain (Table S3). *Centaurea solstitialis* plants from the two native regions, Turkey and Spain, produced from 60% up to 80% more inflorescences compared with plants from Argentina and Chile (*p* < 0.005) (Table S3). Individuals from Turkey, Spain, Argentina, Chile, and Australia produced seeds that were 56%, 42%, 16%, 38%, and 21% lighter compared with individuals from California (*p* < 0.005). In addition, individuals from Spain also presented 22% lighter seeds compared to individuals in Argentina (*p* < 0.005) (Table S3). In the case of days to first flower and length of largest spine, post hoc tests showed no significant differences between the different pairs of regions tested (*p* > 0.05). In a global correlation analysis, several traits demonstrated moderate but significant correlations among each other (Figure S1). Plant height decreased with increasing days to bolting (*r* = −0.25, *p* < 0.001) and days to first flower (*r* = −0.17, *p* = 0.01). Another negative correlation was observed between spine size and days to bolting (*r* = −0.23, *p* < 0.001) and days to first flower (*r* = −0.33, *p* < 0.001). Seed mass increased with the plant height (*r* = 0.13, *p* = 0.05) and spine size (*r* = 0.24, *p* < 0.001) and decreased with increasing the number of capitula (*r* = −0.22, *p* < 0.001) and days to first flower (*r* = −0.26, *p* < 0.001). Number of capitula produced was positively correlated with the plant height (*r* = 0.37, *p* < 0.001) (Figure S1).

### 3.2 Environment characterization and associations with the phenotypic traits

**FIGURE 3:**
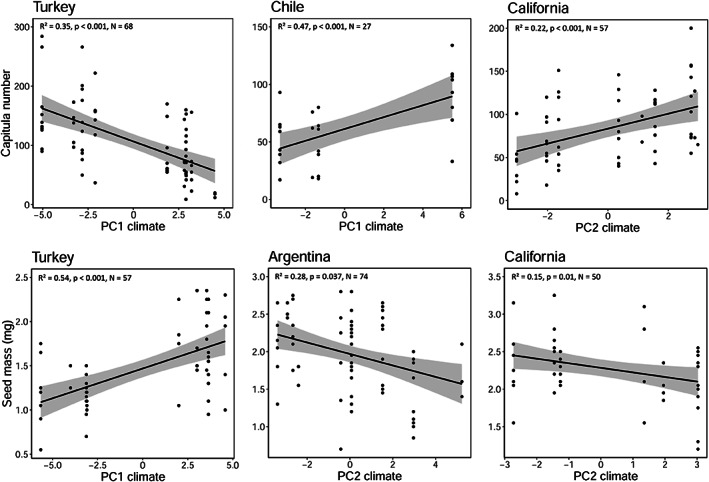
Capitula number and seed size variation along climatic gradients in Turkey (native range) and Argentina, Chile, and California (introduced range).

The PCA on 19 bioclimatic variables showed distinct clustering among regions in terms of climatic space and explained 57.5% variation in the first two PCs axes (Figure S2). Plants in the two native regions experience a higher temperature seasonality (bio4). By comparison, individuals in the introduced regions experience higher annual mean temperatures (bio1) and mean diurnal ranges (bio2), and different precipitation patterns: a wet summer in Argentina (bio18) and Australia (bio14, bio17, bio18), a wet cold season in California (bio16 and bio19), and a high precipitation seasonality in Chile and California (bio 15) (Figure S3). Because we found little overlap in several bioclimatic factors across different geographic regions, we conducted a regional analysis to test for associations between the phenotypic traits and the climate within each region. In Turkey, capitula number showed a negative association with PC1 (Figure 3, Table S4a), which was mainly correlated to the winter temperature and the mean temperature of the wettest quarter (bio11, bio6, bio8, variables that showed negative loadings on the PC1) and to the precipitation of the warmest quarter (bio18, positive loadings on the PC1) (Table S5). Spanish individuals showed no relationship with the climate variables for any of the reproductive traits. In addition, we found no association between the climate and capitula number in Argentinean and Australian individuals (Table S4a). In Chile, capitula number was positively associated with PC1 (Figure 3, Table S4a), which was mainly correlated to the mean temperature of the warmest quarter and mean temperature of the driest quarter as well as to the annual mean temperature and maximum temperature of the warmest month (bio10, bio9, bio1, bio5, all showing negative loadings on the PC1) and to isothermality and precipitation of the driest quarter (bio3, bio17, both showing positive loadings on the PC1) (Table S5). Lastly, capitula number of Californian individuals was positively associated with PC2 (Figure 3, Table S4a), which was mainly correlated to the winter temperature and the mean temperature of the wettest quarter (bio6, bio8, bio11, positive loadings on the PC2) and to mean diurnal range (bio2, negative loading on PC2) (Table S5). Seed mass of Turkish individuals showed a positive association with PC1 (Figure 3, Table S4b). In Argentina, seed mass was negatively associated with PC2 (Figure 3, Table S4b), which was mainly correlated to the maximum temperature of the warmest month and precipitation seasonality (bio5, bio15, both showing positive loadings on the PC2) and to winter precipitation and the precipitation of the driest quarter (bio19, bio17, both showing negative loadings on the PC2) (Table S5). We found no association between seed mass and climate in Chilean individuals. Seed mass of Californian individuals was negatively associated with PC2 (Table S4b).

### 3.3 Candidate outlier SNPs detection

Of 2138 SNP markers, 163 were identified as putative outliers. Three outlier SNPs were identified by all three methods. Another three outliers were identified both by BayeScan and OutFLANK and one outlier both by BayeScan and PCAdapt. Lastly, 12 outliers were identified both by OutFlank and PCAdapt. Consequently, 19 SNPs in total were identified by more than one method. The remaining 144 loci were identified by one method only as following: BayeScan, 4 loci; OutFLANK, 18, and PCAdapt, 122 (Figure S4, Table S6).

### 3.4 Genetic diversity

In general, genetic diversity was similar across native and introduced ranges in terms of allelic richness, observed heterozygosity, and expected heterozygosity. In the non‐native range, populations from Argentina displayed a trend towards increased effective population size, whereas populations from Chile exhibited a trend towards a decrease in N_e_. Moreover, Spain, California and Australia demonstrated a trend towards an increase in inbreeding coefficients compared to the rest of the regions (Table 1). Pairwise region comparison of *F*_ST_ calculated from 1975 neutral genome‐wide SNPs markers revealed low to moderate genetic differentiation between different *C*. *solstitialis* regions, ranging from 0.02 to 0.09 (CI: 0.009–0.11). Pairwise comparisons between Turkey and all other regions demonstrated the highest values of *F*_ST_, whereas genetic differentiation between Spain and all the introduced regions was relatively low (Table 2).

**TABLE 1:** Summary statistics calculated based on 1975 neutral single nucleotide polymorphism loci of *C*. *solstitialis* in the native (Turkey and Spain) and non‐native ranges (Argentina, Chile, California, and Australia).

Region	Range	N	A_r_	H_o_	H_e_	N_e_	F_IS_
Turkey	native	24	1.76	0.17	0.18	54.8	0.07
Spain	native	24	1.83	0.17	0.19	53.0	0.10
Argentina	non‐native	24	1.86	0.18	0.19	177.5	0.05
Chile	non‐native	24	1.80	0.18	0.19	16.8	0.03
California	non‐native	24	1.85	0.17	0.19	33.7	0.11
Australia	non‐native	24	1.81	0.15	0.18	34.8	0.13

Abbreviations: A_r_, allelic richness; F_IS_, inbreeding coefficient; H_e_, expected heterozygosity; H_o,_ observed heterozygosity for polymorphic loci; *N*, number of individuals analyzed; N_e_, effective population size.

**TABLE 2:** Pairwise comparisons of *F*_ST_ values and their 95% lower and upper CI calculated at 1975 neutral SNP loci.

Region	Turkey	Spain	Argentina	Chile	California
Spain	0.087 (0.06–0.11)	‐	‐	‐	‐
Argentina	0.078 (0.05–0.10)	0.029 (0.01–0.04)	‐	‐	‐
Chile	0.089 (0.06–0.11)	0.042 (0.02–0.06)	0.027 (0.01–0.04)	‐	‐
California	0.064 (0.04–0.08)	0.036 (0.02–0.05)	0.022 (0.01–0.03)	0.035 (0.02–0.05)	‐
Australia	0.087 (0.06–0.11)	0.037 (0.02–0.05)	0.021 (0.00–0.03)	0.033 (0.02–0.05)	0.025 (0.01–0.04)

### 3.5 Population genetic structure

**FIGURE 4:**
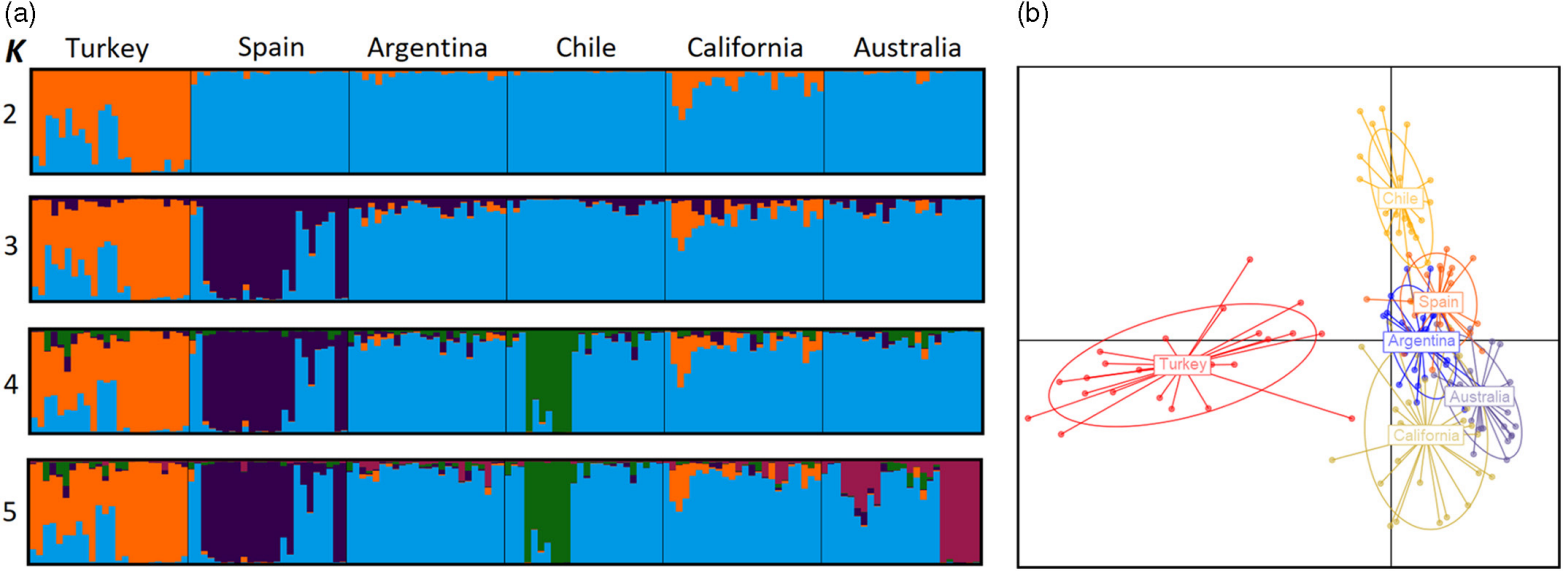
(a) Individual assignments from STRUCTURE analysis based on 1975 neutral SNP loci of 144 individuals of *C*. *solstitialis*. Each vertical bar shows the proportional representation of the estimated group membership for a single individual. *K* is the number of genetic groups. The best estimate of *K* is *K* = 2. (b) Discriminant analysis of principal components (DAPC) based on neutral SNPs and using geographic regions as prior clusters. Ovals are 95% inertia ellipses. Lines connect each individual to the regional mean value.

STRUCTURE analysis identified *K* = 2 as the most probable number of genetic clusters, although some further substructure across regions is also evident (Table S7, Figure S5). In the native area, individuals from Turkey were differentiated from those in Spain, although some individuals in Turkey were not assigned with high certainty and exhibited mixed ancestry. A second genetic group defined Spain and the rest of the invasive range with individuals from California showing slightly higher residual assignment to Turkey than Argentina, Chile and Australia (Figure 4a). Discriminant analysis of principal components revealed population structuring in the native range and offered support for the presence of two genetic groups (Figure 4b), similar to the results of STRUCTURE analysis (Figure 4a). Individuals from native Turkey were separated from all other regions, forming an independent group, whereas individuals from native Spain and the remaining four introduced geographic areas were less distinct and showed overlap.

### 3.6 *P*_ST_‐*F*_ST_ divergence index

For the native versus non‐native comparison, phenotypic trait differentiation (*P*_ST_) exceeded neutral genetic differentiation at SNP loci (*F*_ST_), for two traits related to reproductive success, including seed mass (*P*_ST_ = 0.18) and number of capitula (*P*_ST_ = 0.25) (Table 3). These same two traits also showed climatic associations in some regions (Table S4). In the pairwise *P*_ST_‐*F*_ST_ comparisons, no differences were observed between regions in terms of days to bolting, days to flowering, final plant height, spine length, or capitula number (Table S8). *P*_ST_ values for seed mass exceeded *F*_ST_ values between California and the two native regions (Turkey and Spain) as well as between California and non‐native Chile and Australia, with California showing considerably larger seeds compared with these regions. In addition, differences in seed mass were also found between Argentinean and Turkish populations (Table S8).

**TABLE 3:** Phenotypic and neutral genetic differentiation (*P*_ST_–*F*_ST_ comparison) between the native and non‐native ranges at six morphological traits in *C*. *solstitialis*. Mean *F*_ST_ between native and non‐native ranges was calculated to be 0.024 (0.01–0.03). Traits showing indication of putative divergent selection are highlighted in bold. The lower and upper bound of the 97.5% confidence interval are given in parenthesis.

Trait	Mean *P*_ST_ (97.5% CI)	*P*_ST_ – *F*_ST_ Bayesian credibility interval (97.5% CI)
Days to bolting	0.001 (0.00–0.01)	−0.02 (−0.02 – (−0.01)
Days to first flower	0.002 (0.00–0.01)	−0.02 (−0.02 – (−0.009)
Final plant height	0.007 (0.00–0.03)	−0.02 (−0.02–0.007)
**Capitula number**	**0.25 (0.18–0.32)**	**0.23 (0.16–0.30)**
**Seed mass**	**0.19 (0.08–0.30)**	**0.16 (0.05–0.27)**
Length of largest spine	0.002 (0.00–0.01)	−0.02 (−0.02 – (−0.01)

## 4 DISCUSSION

Our results add to the evidence of the rapid evolutionary capacity of *C. solstitialis* and other invasive plant species more generally. We identified significant trait divergence for seed size and capitula number between the native and non‐native ranges. These traits also seem to be rapidly developing trait–climate associations across several of their non‐native ranges. We add to knowledge about the genetic diversity of this species across its global range, in particular through expanding sampling in Chile and new sampling in Australia. The genetic evidence supports Western Europe, represented by Spain, as a bridgehead source of other non‐native regions based on limited genetic divergence between Spain and all other non‐native regions studied here.

Our evidence of geographical trait differentiation supports previous findings of increased seed mass in the *C*. *solstitialis* non‐native range compared to the native range (Eren & Hierro, 2021; Graebner et al., 2012; Hierro et al., 2013, 2020) but do not support other evidence that *C*. *solstitialis* had evolved towards larger plant size in the introduced area (Barker et al., 2017; Dlugosch et al., 2015; Eriksen et al., 2012; García et al., 2013; Widmer et al., 2007). The fact that we did not detect differences in plant size may be related to the way we scored this trait by measuring the plant height at senescence. Previous studies have used different proxies for plant size including a morphological index that combines information about the leaf number and leaf size at 5 weeks (Barker et al., 2017; Dlugosch et al., 2015). Others have scored the length and width of the fifth true leaf (Eriksen et al., 2012), the rosette diameter (García et al., 2013), and the fresh weights of seedlings at 2 weeks (Widmer et al., 2007). Some of these studies also involved comparing different geographical regions than ours.

The six regions showed differences in several climatic factors. Notably, native populations in both Turkey and Spain occur in more seasonal environments with populations in Turkey experiencing cold wet winters and hot arid summers and populations in Spain experiencing mild winters and relatively cool summers. On the other hand, non‐native populations tend to experience distinct precipitation patterns. Specifically, populations in central Argentina experience cool dry winters and hot wet summers, whereas populations in central Chile and California coast experience a wet cold season and dry summers. In addition, non‐native populations in southeastern Australia experience wet summers like those in Argentina. A previous study that compared native populations from Eurasia with non‐native populations from western USA (California) found that populations in both ranges tend to occupy a similar climatic niche (Dlugosch et al., 2015). We found several significant correlations between the traits we measured and the 30‐year average climate data. Capitula number showed an association with the winter temperature in both Turkey and California, with more capitula being produced in habitats with moderate winters. In Chile, capitula number increased with decreasing summer aridity. Soil water is a known limiting factor in *C*. *solstitialis* invasion (Dlugosch et al., 2015). The species emerges in the fall and overwinters as a basal rosette. Higher water availability, coupled with moderate winter temperatures, could provide an advantage in terms of a faster growth and higher reproductive output (i.e. larger plants producing more capitula, as suggested by the positive correlation we found between these two traits in our study). Similarly, seed mass showed distinct climatic associations in different regions. Seed mass was negatively related to precipitation seasonality in both Turkey and Argentina, with smaller seeds found in highly seasonal habitats. In Argentina, seed mass decreased with a decrease in the winter precipitation and an increase in aridity. In California, seed mass decreased with a decrease in the winter temperature. Our results suggest that different selection pressures in terms of climatic abiotic factors could act on *C*. *solstitialis* seed size in different regions. In other invasive species such as *Echium plantagineum*, populations sourced from hot, arid sites produced heavier seeds than populations from wetter sites, as a strategy to ensure reproductive success in arid environments (Konarzewski et al., 2012). This does not seem to be the case in our study. Hierro et al. (2020) reported the existence of elevation clines in seed size in *C*. *solstitialis* for some of the regions they tested including Caucasus and Anatolia, Western Europe, and Western USA. They showed that seeds at higher elevation were larger than seeds at lower elevation but, also, that seeds in the non‐native regions tended to be larger (i.e., high‐elevation seeds in Eurasia were smaller than high‐elevation seeds in the Americas). Their study supports the hypothesis of a steady increase in seed mass as *C. solstitialis* expanded its distribution across the globe (Hierro et al., 2020) which is coherent with our finding of smaller seeds in the native regions where they generally experience higher seasonal variability. Larger seeds usually produce larger seedlings, which is positively associated with survival, increased competitive ability, and the capacity to withstand different hazards (i.e., herbivory, low nutrients, pathogen attack) which is likely to confer competitive advantages in the non‐native regions (Coomes & Grubb, 2003; Hierro et al., 2013).

Levels of genetic diversity were similar between ranges as shown before by previous molecular studies on *C*. *solstitialis* (Barker et al., 2017; Eriksen et al., 2014). Our estimates of effective population size were somewhat lower in Chile than in other regions, suggesting that this region might have experienced a founder effect population bottleneck following introductions from Spain (Eriksen et al., 2014; Hierro et al., 2009) or it might reflect smaller current population size. Some of the individuals in Chile showed residual assignments to Turkey, suggesting that populations in Asia Minor might have also been contributors to the colonization of South America (see also Eriksen et al., 2014). However, additional simulations of genetic data are needed to test this hypothesis. Populations in Chile occur in small patches, at low densities, and have a slow spread and reduced impact that contrasts with highly invasive populations in Argentina and California (Andonian et al., 2011). Additionally, our observation of no increase in inbreeding coefficient in invasive regions suggests that no shifts in reproductive system from outcrossing towards selfing have occurred across the introduced range of *C. solstitialis,* supporting previous findings (Sun & Ritland, 1998). Genetic differentiation across regions was moderate (paired region *F*_ST_ from 0.021 to 0.089), resulting in the discrimination of two major genetic groups separating native populations in Turkey from the remaining regions. Compared to other short‐lived outcrossing species, these *F*_*S*T_ statistics fall towards the lower end of measures of highly homogenized populations of *Lupinus texensis* (*F*_ST_ from 0.0007 to 0.018) versus populations with an intact native structure such as *Nigella degenii* (*F*_ST_ from 0.03 to 0.07) and *Clarkia xantiana* ssp*. xantiana* (*F*_ST_ from 0.048 to 0.171) (reviewed in Turner et al., 2018). STRUCTURE and DAPC results at neutral SNPs showed limited genetic substructure in the introduced range, consistent with recent colonization and in line with previous results (Barker et al., 2017; Eriksen et al., 2014). Furthermore, we identified three top candidate SNPs (detected by all three methods), suggesting adaptive genomic basis in this species. Given that the RADSeq technology covers only a small fraction of the genome, future whole genome studies would reveal the full extent of the adaptive genome component. Our study is the first to generate molecular markers data for *C*. *solstitialis* populations in Australia. *Centaurea solstitialis* was first reported in Australia around 1856, although the circumstance of its introduction there remains unknown (Parsons & Cuthbertson, 2001). Our genetic structuring analyses showed that Australia was part of the same genetic group comprising native Spain and the rest of the remaining non‐native regions. This indicates that Australia may have been colonized from seeds of Spanish origin or from elsewhere in the American‐invaded range. Individuals from Australia exhibited similarly larger seeds with a pappus to those in Argentina. Future simulation modeling of genetic data might be able to shed further light on the invasive spread of *C. solstitialis* to Australia.

For the range comparison, *P*_ST_ estimates overlapped *F*_ST_ for most of the phenotypic traits investigated_,_ except for seed mass and capitula number, which showed greater *P*_ST_ values than the neutral genetic differentiation. This suggests a role of divergent natural selection in shaping the morphological variation for these traits. Interestingly, a previous *Q*_ST_–*F*_ST_ comparison of *C*. *solstitialis* genotypes from two native (Republic of Georgia and Turkey) and two introduced regions (Argentina and California) found evidence of selection for increased plant size and leaf length in the non‐native range (Eriksen et al., 2012). Evidence of trait divergence in *C*. *solstitialis* from the literature is mixed, with some studies reporting no differences in plant size between native and non‐native ranges (Andonian et al., 2011; Eren & Hierro, 2021; Hierro et al., 2006), while others showed increased growth and higher aboveground biomass in *C*. *solstitialis* introduced range (Dlugosch et al., 2015; Eriksen et al., 2012; García et al., 2013; Graebner et al., 2012; Montesinos & Callaway, 2018a, 2018b; Widmer et al., 2007). These contrasting patterns might be due to natural variability present within different populations of *C*. *solstitialis* in native and/or invasive regions such as genetic drift during early stages of introduction or reflect variable trait expression (phenotypic plasticity) or maternal effects under different environmental conditions used by the different studies (Colautti & Lau, 2015). Nonetheless, our significant *P*_*ST*_ *–F*_*ST*_ results support a hypothesis of selective divergence in seed mass as well as previous observations that seed mass is putatively an adaptive trait in some of the species' introduced ranges (Hierro et al., 2011, 2013, 2020). Likewise, *P*_ST_–*F*_ST_ analysis at the paired regional level indicated divergent selection for increased seed mass in non‐native California compared to the two native regions (Turkey and Spain) as well as compared to non‐native Chile and Australia and increased seed mass in non‐native Argentina compared to native Turkey. Our study focused only on seed mass of the more abundant pappus‐bearing seeds, but the fact that Hierro et al. (2020) reported a nearly parallel variation in seed size for both pappus and non‐pappus seeds suggests that the results could be also a good proxy for non‐pappus seeds. Admixture events in this species may also be an explanation for the observed increases in seed size. Both historical and genetic studies point towards a complex invasion history with multiple introductions from different sources that could have created optimum conditions for enhanced plant performance and heterosis (Barker et al., 2017; Eriksen et al., 2014). Nonetheless, admixture benefits seem to decrease as the genetic divergence between parents increases as exemplified by previous studies on *C*. *solstitialis* that performed experimental crosses between geographically distinct populations and measured plant growth, competitive ability, and seed production (Barker et al., 2019; Irimia et al., 2021; Montesinos & Callaway, 2018a). As for the capitula number, *P*_*ST*_ estimates seem to have been influenced mostly by the low values present in populations in South America. It is unclear to us why native populations are more fecund. Presumably, this could be related to distinct plant reproductive strategies in different ranges and habitats or perhaps genetic drift related to the founder effect or reduced Ne detected for Chile. Specifically, another study found that native individuals in Anatolia have smaller capitulum size and produce more seeds without a pappus compared to non‐native individuals in Argentina which showed the opposite pattern (i.e. larger capitula and more pappus seeds) (Eren & Hierro, 2021). According to Miguel et al. (2017), seed dimorphisms in *C*. *solstitialis* relate to a task division strategy in which pappus seeds are involved in dispersal and colonization of new habitats, whereas non‐pappus seeds ensure site persistence. Both capitula number and seed size showed a positive relationship with plant height suggesting no trade‐off between growth and reproduction. The evolution of increased biomass (Barker et al., 2017) and competitive abilities (Hierro et al., 2022) previously reported in this species, combined with the evolution of seed size that we found in our study could represent an important part of *C*. *solstitialis* invasion success.

Our results on trait evolution need to be regarded cautiously since we used seeds collected in the field, thus, we cannot exclude environment of origin effects on the phenotypic traits we measured. However, we argue that plant maternal effects are probably minor in *C*. *solstitialis*, at least for reproductive traits. Maternal effects seem to be more prevalent at the initial plant stages and seed weight appears to be one of the least plastic plant traits (Hierro et al., 2020). Although desirable, comprehensive sampling was not possible within all regions with *C*. *solstitialis* presence (i.e. Caucasus, Eastern Europe, and Pacific Northwest). Therefore, the findings and conclusions that we present here are limited to the regions sampled. More extensive sampling within existing regions would better detect variation at these scales. Despite the extra preparation, we recommend that future research should aim to measure glasshouse‐produced seed from controlled crosses to better control for potential maternal effects. Nonetheless, despite all these challenges, our significant results based on *P*_ST_ Bayesian 97.5% credibility intervals (Leinonen et al., 2013) are still informative about the traits putatively under divergent selection.

Overall, our findings suggest that genetic drift is unlikely to be the sole force behind the observed biogeographic phenotypic variation in *C*. *solstitialis*. Evidence consistent with adaptive rapid evolution in phenotypic traits in the introduced range exemplifies how different environmental conditions across world regions result in unique local evolutionary and ecological dynamics within each region. Our results might be relevant to the management of this invasive weed, indicating that management practices should try to prevent or reduce yellow starthistle seed production and seed bank, thereby limiting further local adaptation.

## CONFLICT OF INTEREST STATEMENT

The authors declare that they have no conflict of interest.

## Supporting information


Appendix S1

## Data Availability

Sequencing data for each individual can be found on NCBI, BioProject ID: PRJNA950038.
