# Supplementary material for: Trait evolution during a rapid global weed invasion despite little genetic differentiation
Source: Evol Appl. 2023 Apr 18;16(5):997–1011. doi: 10.1111/eva.13548 (PMC10197227; doi:10.1111/eva.13548)
Supplement: Supplementary file 1 — Appendix S1 [file EVA-16-997-s001.docx]

**Supplementary Information**

**Trait evolution during a rapid global weed invasion despite little genetic differentiation**

**Running title:** Genetics of colonizing and invasive species

Ramona E. Irimia^1,2^; Daniel Montesinos^1,3^, Anurag Chaturvedi^4,5^, Ian Sanders^4^, José L. Hierro^6,7^; Gastón Sotes^6^; Lohengrin A. Cavieres^8,9^; Özkan Eren^10^; Christopher J. Lortie^11,12^; Kristine French^13^, Adrian Christopher Brennan^14*^

*^1^Centre for Functional Ecology, Department of Life Sciences, University of Coimbra, Coimbra, Portugal*

*^2^ Plant Evolutionary Ecology, Institute of Evolution and Ecology, University of Tübingen, Tübingen, Germany*

*^3^Australian Tropical Herbarium, James Cook University, Cairns, Queensland, Australia*

*^4^Department of Ecology and Evolution, University of Lausanne, Lausanne, Switzerland*

*^5^Environmental Genomics Group, School of Biosciences, University of Birmingham, Birmingham, UK*

*^6^Laboratorio de Ecología, Biogeografía y Evolución Vegetal (LEByEV), Instituto de Ciencias de la Tierra y Ambientales de La Pampa (INCITAP), Consejo Nacional de Investigaciones Científicas y Técnicas (CONICET), Universidad Nacional de La Pampa (UNLPam), Santa Rosa, Argentina*

*^7^Departamento de Biología, Facultad de Ciencias Exactas y Naturales, UNLPam, Santa Rosa, Argentina*

*^8^Departamento de Botánica, Facultad de Ciencias Naturales y Oceanográficas, Universidad de Concepción, Concepción, Chile*

*^9^Instituto de Ecología y Biodiversidad (IEB), Santiago, Chile*

*^10^Aydın Adnan Menderes Üniversitesi, Biyoloji Bölümü, Fen-Edebiyat Fakültesi, Aydın, Turkey*

*^11^Department of Biology, York University, Toronto, Canada*

*^12^ The National Center for Ecological Analysis and Synthesis (NCEAS), UCSB, Santa Barbara, California, USA*

*^13^School of Earth, Atmospheric and Life Sciences, University of Wollongong, Wollongong, Australia*

*^14^School of Biological and Biomedical Sciences, University of Durham, Durham, UK*

*Corresponding author: Adrian Christopher Brennan

E-mail: a.c.brennan@durham.ac.uk

**Table S1.** GPS coordinates of the *C*. *solstitialis* populations sampled in this study (Datum WGS84). N = the number of individuals sampled from each population for phenotypic analysis. For genetic analysis, only a subset of these individuals was used (ie. 144 individuals, 24 individuals from each of the six regions).

| **Pop** | **Region** | **N** | **Province/County** | **Location** | **Latitude** | **Longitude** | **Altitude (m)** | **Collection Date** |
| --- | --- | --- | --- | --- | --- | --- | --- | --- |
| 1 | Turkey | 10 | Denizli | Pınarkent | 37.80283 | 29.19525 | 262 | 2013 |
| 2 | Turkey | 6 | Afyonkarahisar | Dazkırı | 37.95083 | 29.84033 | 929 | 2013 |
| 3 | Turkey | 6 | Isparta | Isparta | 37.89844 | 30.43828 | 939 | 2013 |
| 4 | Turkey | 7 | Burdur | Burdur | 37.61608 | 30.14617 | 923 | 2013 |
| 5 | Turkey | 7 | Denizli | Serinhisar | 37.53156 | 29.30086 | 871 | 2013 |
| 6 | Turkey | 5 | Afyonkarahisar | Dinar to Çay | 38.15392 | 30.23417 | 1040 | 2013 |
| 7 | Turkey | 10 | Izmir | Beydağ | 38.08586 | 28.21547 | 214 | 2013 |
| 8 | Turkey | 9 | Izmir | Bozdağ | 38.30136 | 28.04986 | 1189 | 2013 |
| 9 | Turkey | 5 | Aydın | Akçaköy | 37.95292 | 28.02997 | 501 | 2013 |
| 10 | Turkey | 6 | Aydın | Geyre | 37.71231 | 28.69269 | 477 | 2013 |
| 1 | Spain | 9 | Cuenca | Moncalvillo de Huete | 40.24159 | -2.687453 | 736 | 2009 |
| 2 | Spain | 5 | Tarragona | Batea | 41.06882 | 0.334305 | 368 | 2009 |
| 3 | Spain | 10 | Lleida | L´Espluga-Calba | 41.50499 | 1.005857 | 493 | 2009 |
| 4 | Spain | 8 | Lleida | Gerb | 41.87776 | 0.778373 | 583 | 2009 |
| 5 | Spain | 7 | Cuenca | Villares del Sanz | 39.84719 | -2.501784 | 872 | 2014 |
| 6 | Spain | 10 | Salamanca | Castellanos de Moriscos | 41.02614 | -5.605175 | 814 | 2014 |
| 7 | Spain | 10 | Burgos | La Horra | 41.7288 | -3.834349 | 736 | 2014 |
| 8 | Spain | 6 | Valladolid | Castronuño | 41.39246 | -5.276957 | 698 | 2014 |
| 9 | Spain | 6 | Cuenca | Tarancón | 40.01424 | -2.9731 | 736 | 2014 |
| 10 | Spain | 6 | Zaragoza | Sástago | 41.40823 | -0.289773 | 324 | 2014 |
| 1 | Chile | 4 | Talagante | Padre Hurtado | -33.57083 | -70.85528 | 395 | 2013 |
| 2 | Chile | 9 | Talagante | El Monte | -33.68944 | -71.05528 | 239 | 2013 |
| 3 | Chile | 7 | Santiago | Maipú | -33.52472 | -70.75167 | 479 | 2013 |
| 4 | Chile | 8 | Santiago | Lo Barnechea | -33.37 | -70.42972 | 1123 | 2013 |
| 1 | Argentina | 9 | La Pampa | Paraje El Tropezón, R14 | -36.709 | -64.83106 | 294 | 2013 |
| 2 | Argentina | 7 | La Pampa | El Durazno, R14, km 189 | -36.70008 | -65.39142 | 338 | 2013 |
| 3 | Argentina | 11 | La Pampa | Rucanelo, R11 y R10 | -36.70894 | -64.83083 | 294 | 2013 |
| 4 | Argentina | 9 | La Pampa | Victorica | -36.25011 | -65.45425 | 304 | 2013 |
| 5 | Argentina | 7 | La Pampa | Winifreda | -36.22397 | -64.28133 | 159 | 2013 |
| 6 | Argentina | 9 | La Pampa | Santa Rosa | -36.61658 | -64.25625 | 188 | 2013 |
| 7 | Argentina | 9 | La Pampa | Trenel, R35 km 426 | -35.72458 | -64.26928 | 192 | 2013 |
| 8 | Argentina | 9 | La Pampa | Quehué, R 35 y R18 | -37.12161 | -64.28661 | 199 | 2013 |
| 9 | Argentina | 8 | La Pampa | Unanue, R35 km 215 | -37.55967 | -64.2915 | 226 | 2013 |
| 10 | Argentina | 8 | La Pampa | Lonquimay, R1 km 203 | -36.56553 | -63.66467 | 138 | 2013 |
| 1 | California | 11 | Sacramento | Folsom | 38.64215 | -121.176 | 72 | 2009 |
| 2 | California | 1 | Sacramento | Folsom | 38.68293 | -121.1811 | 48 | 2009 |
| 3 | California | 2 | Marin | Novato | 38.15622 | -122.6926 | 125 | 2009 |
| 4 | California | 8 | Solano | Green Valley | 38.20954 | -122.1463 | 20 | 2009 |
| 5 | California | 9 | Sonoma | Petaluma | 38.22456 | -122.5341 | 74 | 2009 |
| 6 | California | 2 | Sonoma | Petaluma | 38.23643 | -122.5638 | 43 | 2009 |
| 7 | California | 9 | Napa | Napa | 38.33904 | -122.1547 | 88 | 2009 |
| 8 | California | 7 | Solano | Vacaville | 38.41059 | -121.9343 | 27 | 2009 |
| 9 | California | 9 | Napa | Napa | 38.45353 | -122.1529 | 189 | 2009 |
| 1 | Australia | 4 | NSW | Colbinalbin | -36.59549 | 144.7363 | 190 | 2012 |
| 2 | Australia | 6 | NSW | Hume, 5km N of Holbrook | -36.67779 | 147.3697 | 1200 | 2012 |
| 3 | Australia | 7 | NSW | Gundagai | -35.0672 | 148.1085 | 211 | 2012 |
| 4 | Australia | 8 | NSW | 18 km N of Cudal | -33.22193 | 148.9088 | 625 | 2014 |
| 5 | Australia | 8 | NSW | Koorawatha | -34.01678 | 148.569 | 298 | 2014 |
| 6 | Australia | 8 | NSW | Murringo | -34.31917 | 148.4932 | 436 | 2014 |
| 7 | Australia | 9 | NSW | Yass | -34.87395 | 148.9083 | 501 | 2014 |

**Table S2.** Adapters sequences used for ddRADseq experiment were kindly provided by the Dlugosch lab (see more details in Barker et al. 2017). Asterisks represent modified nucleotides (phosphorothioate modification) to prevent nuclease degradation. Uppercase letters in DNA coding indicate adapter sequence while lower case letters indicate 6bp barcode sequence. N = complementary pairs of oligonucleotides to be annealed and used in the ligation step of DNA library preparation.

| **ID** | **Sequence** | **N** | **Index** |
| --- | --- | --- | --- |
| P1_PstI_top_i1 | AATGATACGGCGACCACCGAGATCTACACTCTTTCCCTACACGACGCTCTTCCGATCTgcatgaTGC*A | 1 | gcatga |
| P1_PstI_bottom_i1 | /5Phos/tcatgcAGATCGGAAGAGCGTCGTGTAGGGAAAGAGTGTAGATCTCGGTGGTCGCCGTATCAT*T | 1 | tcatgc |
| P2_Mse_top_i1 | /5Phos/TAgcatgaAGATCGGAAGAGCGGTTCAGCAGGAATGCCGAGACCGATCAGAACAA | 1 | gcatga |
| P2_Mse_bottom_i1 | CAAGCAGAAGACGGCATACGAGATCGGTCTCGGCATTCCTGCTGAACCGCTCTTCCGATCTtcatg*c | 1 | tcatg |
| P1_PstI_top_i2 | AATGATACGGCGACCACCGAGATCTACACTCTTTCCCTACACGACGCTCTTCCGATCTgatccgTGC*A | 2 | gatccg |
| P1_PstI_bottom_i2 | /5Phos/cggatcAGATCGGAAGAGCGTCGTGTAGGGAAAGAGTGTAGATCTCGGTGGTCGCCGTATCAT*T | 2 | cggatc |
| P2_Mse_top_i2 | /5Phos/TAgatccgAGATCGGAAGAGCGGTTCAGCAGGAATGCCGAGACCGATCAGAACAA | 2 | gatccg |
| P2_Mse_bottom_i2 | CAAGCAGAAGACGGCATACGAGATCGGTCTCGGCATTCCTGCTGAACCGCTCTTCCGATCTcggat*c | 2 | cggat |
| P1_PstI_top_i3 | AATGATACGGCGACCACCGAGATCTACACTCTTTCCCTACACGACGCTCTTCCGATCTtccagtTGC*A | 3 | tccagt |
| P1_PstI_bottom_i3 | /5Phos/actggaAGATCGGAAGAGCGTCGTGTAGGGAAAGAGTGTAGATCTCGGTGGTCGCCGTATCAT*T | 3 | actgga |
| P2_Mse_top_i3 | /5Phos/TAtccagtAGATCGGAAGAGCGGTTCAGCAGGAATGCCGAGACCGATCAGAACAA | 3 | tccagt |
| P2_Mse_bottom_i3 | CAAGCAGAAGACGGCATACGAGATCGGTCTCGGCATTCCTGCTGAACCGCTCTTCCGATCTactgg*a | 3 | actgg |
| P1_PstI_top_i4 | AATGATACGGCGACCACCGAGATCTACACTCTTTCCCTACACGACGCTCTTCCGATCTgtctta TGC*A | 4 | gtctta |
| P1_PstI_bottom_i4 | /5Phos/taagacAGATCGGAAGAGCGTCGTGTAGGGAAAGAGTGTAGATCTCGGTGGTCGCCGTATCAT*T | 4 | taagac |
| P2_Mse_top_i4 | /5Phos/TAgtctta AGATCGGAAGAGCGGTTCAGCAGGAATGCCGAGACCGATCAGAACAA | 4 | gtctta |
| P2_Mse_bottom_i4 | CAAGCAGAAGACGGCATACGAGATCGGTCTCGGCATTCCTGCTGAACCGCTCTTCCGATCTtaaga*c | 4 | taaga |
| P1_PstI_top_i5 | AATGATACGGCGACCACCGAGATCTACACTCTTTCCCTACACGACGCTCTTCCGATCTcggagtTGC*A | 5 | cggagt |
| P1_PstI_bottom_i5 | /5Phos/actccgAGATCGGAAGAGCGTCGTGTAGGGAAAGAGTGTAGATCTCGGTGGTCGCCGTATCAT*T | 5 | actccg |
| P2_Mse_top_i5 | /5Phos/TAcggagtAGATCGGAAGAGCGGTTCAGCAGGAATGCCGAGACCGATCAGAACAA | 5 | cggagt |
| P2_Mse_bottom_i5 | CAAGCAGAAGACGGCATACGAGATCGGTCTCGGCATTCCTGCTGAACCGCTCTTCCGATCTactcc*g | 5 | actcc |
| P1_PstI_top_i6 | AATGATACGGCGACCACCGAGATCTACACTCTTTCCCTACACGACGCTCTTCCGATCTcacgttTGC*A | 6 | cacgtt |
| P1_PstI_bottom_i6 | /5Phos/aacgtgAGATCGGAAGAGCGTCGTGTAGGGAAAGAGTGTAGATCTCGGTGGTCGCCGTATCAT*T | 6 | aacgtg |
| P2_Mse_top_i6 | /5Phos/TAcacgttAGATCGGAAGAGCGGTTCAGCAGGAATGCCGAGACCGATCAGAACAA | 6 | cacgtt |
| P2_Mse_bottom_i6 | CAAGCAGAAGACGGCATACGAGATCGGTCTCGGCATTCCTGCTGAACCGCTCTTCCGATCTaacgt*g | 6 | aacgt |
| P1_PstI_top_i7 | AATGATACGGCGACCACCGAGATCTACACTCTTTCCCTACACGACGCTCTTCCGATCTatacagTGC*A | 7 | atacag |
| P1_PstI_bottom_i7 | /5Phos/ctgtatAGATCGGAAGAGCGTCGTGTAGGGAAAGAGTGTAGATCTCGGTGGTCGCCGTATCAT*T | 7 | ctgtat |
| P2_Mse_top_i7 | /5Phos/TAatacagAGATCGGAAGAGCGGTTCAGCAGGAATGCCGAGACCGATCAGAACAA | 7 | atacag |
| P2_Mse_bottom_i7 | CAAGCAGAAGACGGCATACGAGATCGGTCTCGGCATTCCTGCTGAACCGCTCTTCCGATCTctgta*t | 7 | ctgta |
| P1_PstI_top_i8 | AATGATACGGCGACCACCGAGATCTACACTCTTTCCCTACACGACGCTCTTCCGATCTtgttacTGC*A | 8 | tgttac |
| P1_PstI_bottom_i8 | /5Phos/gtaacaAGATCGGAAGAGCGTCGTGTAGGGAAAGAGTGTAGATCTCGGTGGTCGCCGTATCAT*T | 8 | gtaaca |
| P2_Mse_top_i8 | /5Phos/TAtgttacAGATCGGAAGAGCGGTTCAGCAGGAATGCCGAGACCGATCAGAACAA | 8 | tgttac |
| P2_Mse_bottom_i8 | CAAGCAGAAGACGGCATACGAGATCGGTCTCGGCATTCCTGCTGAACCGCTCTTCCGATCTgtaac*a | 8 | gtaac |
| P1_PstI_top_i9 | AATGATACGGCGACCACCGAGATCTACACTCTTTCCCTACACGACGCTCTTCCGATCTacgctcTGC*A | 9 | acgctc |
| P1_PstI_bottom_i9 | /5Phos/gagcgtAGATCGGAAGAGCGTCGTGTAGGGAAAGAGTGTAGATCTCGGTGGTCGCCGTATCAT*T | 9 | gagcgt |
| P2_Mse_top_i9 | /5Phos/TAacgctcAGATCGGAAGAGCGGTTCAGCAGGAATGCCGAGACCGATCAGAACAA | 9 | acgctc |
| P2_Mse_bottom_i9 | CAAGCAGAAGACGGCATACGAGATCGGTCTCGGCATTCCTGCTGAACCGCTCTTCCGATCTgagcg*t | 9 | gagcg |
| P1_PstI_top_i10 | AATGATACGGCGACCACCGAGATCTACACTCTTTCCCTACACGACGCTCTTCCGATCTttggcaTGC*A | 10 | ttggca |
| P1_PstI_bottom_i10 | /5Phos/tgccaaAGATCGGAAGAGCGTCGTGTAGGGAAAGAGTGTAGATCTCGGTGGTCGCCGTATCAT*T | 10 | tgccaa |
| P2_Mse_top_i10 | /5Phos/TAttggcaAGATCGGAAGAGCGGTTCAGCAGGAATGCCGAGACCGATCAGAACAA | 10 | ttggca |
| P2_Mse_bottom_i10 | CAAGCAGAAGACGGCATACGAGATCGGTCTCGGCATTCCTGCTGAACCGCTCTTCCGATCTtgcca*a | 10 | tgcca |
| P1_PstI_top_i11 | AATGATACGGCGACCACCGAGATCTACACTCTTTCCCTACACGACGCTCTTCCGATCTagtaacTGC*A | 11 | agtaac |
| P1_PstI_bottom_i11 | /5Phos/gttactAGATCGGAAGAGCGTCGTGTAGGGAAAGAGTGTAGATCTCGGTGGTCGCCGTATCAT*T | 11 | gttact |
| P2_Mse_top_i11 | /5Phos/TAagtaacAGATCGGAAGAGCGGTTCAGCAGGAATGCCGAGACCGATCAGAACAA | 11 | agtaac |
| P2_Mse_bottom_i11 | CAAGCAGAAGACGGCATACGAGATCGGTCTCGGCATTCCTGCTGAACCGCTCTTCCGATCTgttac*t | 11 | gttac |
| P1_PstI_top_i12 | AATGATACGGCGACCACCGAGATCTACACTCTTTCCCTACACGACGCTCTTCCGATCTcaagcgTGC*A | 12 | caagcg |
| P1_PstI_bottom_i12 | /5Phos/cgcttgAGATCGGAAGAGCGTCGTGTAGGGAAAGAGTGTAGATCTCGGTGGTCGCCGTATCAT*T | 12 | cgcttg |
| P2_Mse_top_i12 | /5Phos/TAcaagcgAGATCGGAAGAGCGGTTCAGCAGGAATGCCGAGACCGATCAGAACAA | 12 | caagcg |
| P2_Mse_bottom_i12 | CAAGCAGAAGACGGCATACGAGATCGGTCTCGGCATTCCTGCTGAACCGCTCTTCCGATCTcgctt*g | 12 | cgctt |
| P1_PstI_top_i13 | AATGATACGGCGACCACCGAGATCTACACTCTTTCCCTACACGACGCTCTTCCGATCTatgctaTGC*A | 13 | atgcta |
| P1_PstI_bottom_i13 | /5Phos/tagcatAGATCGGAAGAGCGTCGTGTAGGGAAAGAGTGTAGATCTCGGTGGTCGCCGTATCAT*T | 13 | tagcat |
| P2_Mse_top_i13 | /5Phos/TAatgctaAGATCGGAAGAGCGGTTCAGCAGGAATGCCGAGACCGATCAGAACAA | 13 | atgcta |
| P2_Mse_bottom_i13 | CAAGCAGAAGACGGCATACGAGATCGGTCTCGGCATTCCTGCTGAACCGCTCTTCCGATCTtagca*t | 13 | tagca |

**Figure S1**. Correlation between traits. The boxes in diagonal display the density plot for each variable as well as the bar plot with the number of observations for each group. The boxes above the diagonal display the Pearson correlation. The value of the correlation and the significance level is indicated by asterisks (0.1 = “.”, 0.05 = “*”, 0.01 = “**”, 0.001 = (“***”). The boxes below the diagonal display the scatterplot between each variable and the histograms (at the bottom) for each group. The variable names are displayed on the outer edge of the matrix. Box plots on the right show the median for each variable. Region abbreviation: AR = Argentina, AU = Australia, CA = California, CL = Chile, SP = Spain and TR = Turkey.


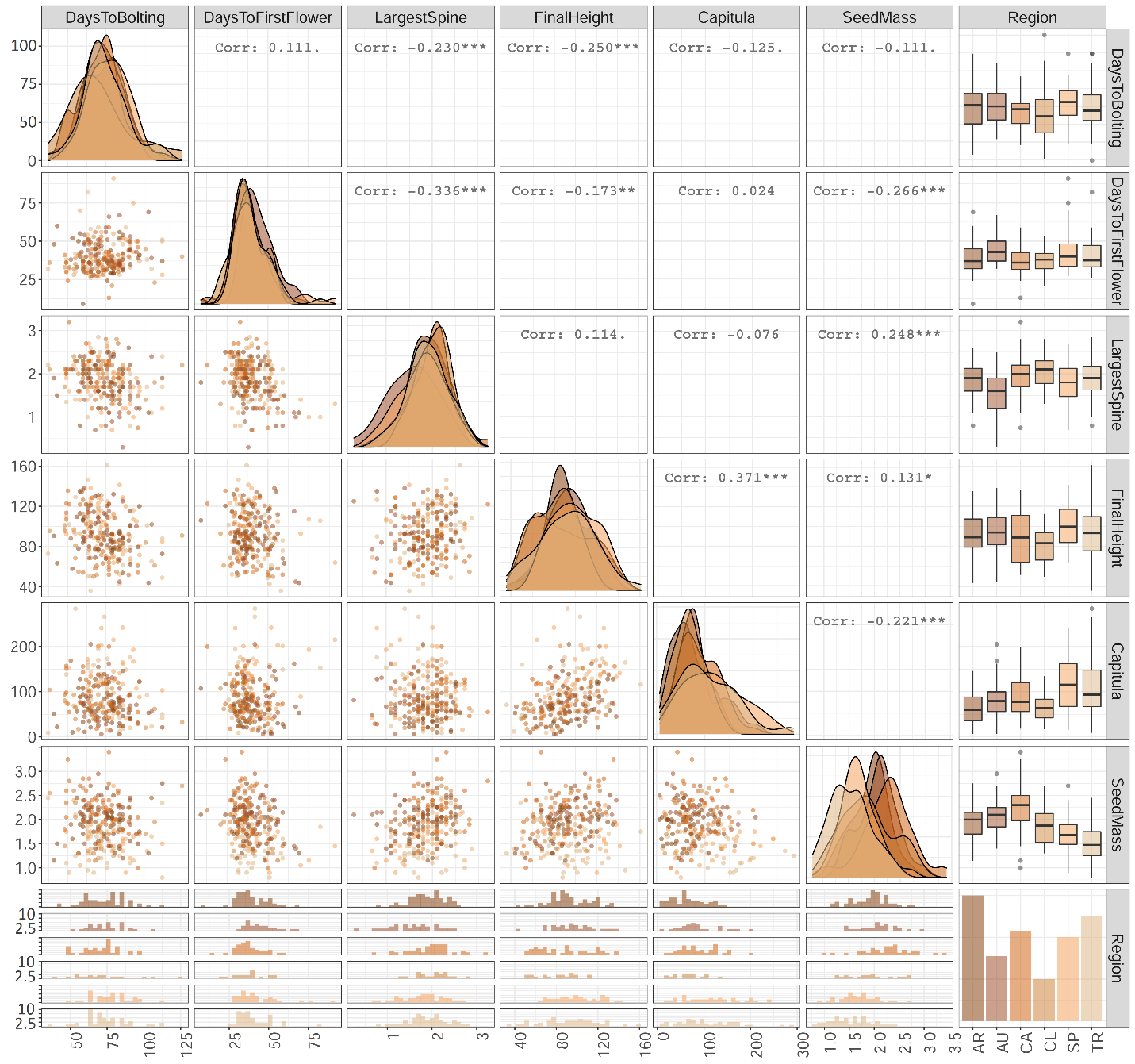


**Table S3.** Tukey post hoc test output for comparison of traits mean of *C*. *solstitialis* between regions. Z value = measure of standard deviation, Pr = probability that the null hypothesis has been rejected.

| **Trait** | **Comparison** | **Estimate** | **Std. Error** | **Z value** | **Pr (>\|z\|)** |
| --- | --- | --- | --- | --- | --- |
|  |  |  |  |  |  |
| **Days to bolting** | Spain vs Turkey | -3.32 | 3.46 | -0.95 | 0.92 |
|  | Argentina vs Turkey | -0.94 | 3.53 | -0.26 | 0.99 |
|  | Chile vs Turkey | -3.35 | 4.73 | -0.70 | 0.98 |
|  | California vs Turkey | 11.4 | 5.01 | 2.29 | 0.18 |
|  | Australia vs Turkey | -2.59 | 3.91 | -0.66 | 0.98 |
|  | Argentina vs Spain | 2.37 | 3.34 | 0.70 | 0.97 |
|  | Chile vs Spain | -0.03 | 4.93 | -0.00 | 1.00 |
|  | California vs Spain | 14.8 | 4.76 | 3.10 | **0.02*** |
|  | Australia vs Spain | 0.73 | 3.79 | 0.19 | 1.00 |
|  | Chile vs Argentina | 2.40 | 4.78 | 0.50 | 0.99 |
|  | California vs Argentina | -12.42 | 4.75 | -2.61 | 0.09. |
|  | Australia vs Argentina | 1.64 | 3.69 | 0.44 | 0.99 |
|  | California vs Chile | 14.8 | 6.08 | 2.44 | 0.13 |
|  | Australia vs Chile | 0.76 | 5.12 | 0.14 | 1.00 |
|  | Australia vs California | -14.07 | 5.07 | -2.77 | 0.058. |
| **Days to first flower** | Spain vs Turkey | -5.25 | 2.90 | -1.80 | 0.44 |
|  | Argentina vs Turkey | 0.90 | 2.91 | 0.31 | 0.99 |
|  | Chile vs Turkey | 3.63 | 4.03 | 0.90 | 0.94 |
|  | California vs Turkey | 0.83 | 4.13 | 0.20 | 1.00 |
|  | Australia vs Turkey | -7.14 | 3.23 | -2.20 | 0.22 |
|  | Argentina vs Spain | 6.16 | 2.78 | 2.21 | 0.22 |
|  | Chile vs Spain | 8.89 | 4.22 | 2.10 | 0.27 |
|  | California vs Spain | 6.09 | 3.94 | 1.54 | 0.62 |
|  | Australia vs Spain | -1.88 | 3.16 | -0.59 | 0.99 |
|  | Chile vs Argentina | -2.73 | 4.07 | -0.67 | 0.98 |
|  | California vs Argentina | 0.07 | 3.89 | 0.01 | 1.00 |
|  | Australia vs Argentina | 8.04 | 3.04 | 2.64 | 0.08. |
|  | California vs Chile | -2.80 | 5.09 | -0.55 | 0.99 |
|  | Australia vs Chile | -10.78 | 4.30 | -2.50 | 0.11 |
|  | Australia vs California | -7.97 | 4.14 | -1.92 | 0.37 |
| **Capitula** | Spain vs Turkey | -4.065 | 12.7 | -0.31 | 0.99 |
|  | Argentina vs Turkey | 45.2 | 13.0 | 3.47 | **0.006**** |
|  | Chile vs Turkey | 72.0 | 17.4 | 4.12 | **<0.001***** |
|  | California vs Turkey | 15.7 | 18.7 | 0.83 | 0.95 |
|  | Australia vs Turkey | 32.8 | 14.4 | 2.27 | 0.19 |
|  | Argentina vs Spain | 49.3 | 12.3 | 4.00 | **<0.001***** |
|  | Chile vs Spain | 76.1 | 18.2 | 4.16 | **<0.001***** |
|  | California vs Spain | 19.8 | 17.8 | 1.11 | 0.87 |
|  | Australia vs Spain | 36.9 | 14.0 | 2.64 | 0.08 |
|  | Chile vs Argentina | -26.8 | 17.6 | -1.51 | 0.64 |
|  | California vs Argentina | 29.5 | 17.7 | 1.65 | 0.54 |
|  | Australia vs Argentina | 12.3 | 13.6 | 0.91 | 0.94 |
|  | California vs Chile | -56.3 | 22.7 | -2.47 | 0.12 |
|  | Australia vs Chile | -39.2 | 18.9 | -2.06 | 0.29 |
|  | Australia vs California | 17.1 | 18.9 | 0.90 | 0.94 |
| **Seed mass** | Spain vs Turkey | -0.07 | 0.14 | -0.52 | 0.99 |
|  | Argentina vs Turkey | 0.35 | 0.13 | 2.57 | 0.09. |
|  | Chile vs Turkey | 0.15 | 0.20 | 0.72 | 0.97 |
|  | California vs Turkey | 1.01 | 0.19 | 5.31 | **< 0.001***** |
|  | Australia vs Turkey | 0.30 | 0.16 | 1.83 | 0.42 |
|  | Argentina vs Spain | 0.42 | 0.13 | 3.15 | **0.01*** |
|  | Chile vs Spain | 0.22 | 0.22 | 1.00 | 0.91 |
|  | California vs Spain | 1.08 | 0.19 | 5.64 | <**0.001***** |
|  | Australia vs Spain | 0.38 | 0.16 | 2.32 | 0.17 |
|  | Chile vs Argentina | -0.20 | 0.22 | -0.90 | 0.94 |
|  | California vs Argentina | 0.65 | 0.18 | 3.48 | **0.006**** |
|  | Australia vs Argentina | -0.04 | 0.15 | -0.31 | 0.99 |
|  | California vs Chile | 0.86 | 0.25 | 3.31 | **0.01*** |
|  | Australia vs Chile | 0.15 | 0.24 | 0.63 | 0.98 |
|  | Australia vs California | -0.70 | 0.20 | -3.52 | **0.005**** |
| **Largest spine** | Spain vs Turkey | 0.30 | 0.13 | 2.24 | 0.21 |
|  | Argentina vs Turkey | 0.13 | 0.13 | 1.00 | 0.91 |
|  | Chile vs Turkey | -0.04 | 0.18 | -0.25 | 1.00 |
|  | California vs Turkey | 0.05 | 0.17 | 0.32 | 1.00 |
|  | Australia vs Turkey | 0.37 | 0.15 | 2.47 | 0.12 |
|  | Argentina vs Spain | -0.17 | 0.13 | -1.27 | 0.79 |
|  | Chile vs Spain | -0.35 | 0.18 | -1.87 | 0.41 |
|  | California vs Spain | -0.25 | 0.17 | -1.46 | 0.67 |
|  | Australia vs Spain | 0.06 | 0.15 | 0.46 | 0.99 |
|  | Chile vs Argentina | 0.18 | 0.18 | 1.00 | 0.91 |
|  | California vs Argentina | 0.08 | 0.17 | 0.47 | 0.99 |
|  | Australia vs Argentina | -0.23 | 0.14 | -1.62 | 0.57 |
|  | California vs Chile | 0.10 | 0.21 | 0.47 | 0.99 |
|  | Australia vs Chile | 0.42 | 0.19 | 2.14 | 0.25 |
|  | Australia vs California | 0.32 | 0.18 | 1.75 | 0.49 |
| **Final plant height** | Spain vs Turkey | -1.90 | 6.07 | -0.31 | 1.00 |
|  | Argentina vs Turkey | 1.33 | 6.17 | 0.21 | 1.00 |
|  | Chile vs Turkey | 12.04 | 8.25 | 1.45 | 0.68 |
|  | California vs Turkey | 1.34 | 8.36 | 0.16 | 1.00 |
|  | Australia vs Turkey | 2.80 | 6.82 | 0.41 | 0.99 |
|  | Argentina vs Spain | 3.24 | 5.90 | 0.54 | 0.99 |
|  | Chile vs Spain | 13.95 | 8.55 | 1.63 | 0.56 |
|  | California vs Spain | 3.25 | 8.00 | 0.40 | 0.99 |
|  | Australia vs Spain | 4.71 | 6.65 | 0.70 | 0.98 |
|  | Chile vs Argentina | -10.7 | 8.32 | -1.28 | 0.78 |
|  | California vs Argentina | -0.00 | 8.00 | -0.01 | 1.00 |
|  | Australia vs Argentina | -1.46 | 6.52 | -0.22 | 1.00 |
|  | California vs Chile | -10.69 | 10.24 | -1.04 | 0.89 |
|  | Australia vs Chile | -9.24 | 8.90 | -1.03 | 0.90 |
|  | Australia vs California | 1.45 | 8.55 | 0.17 | 1.00 |

**Figure S2**. Scaled and centered PCA on 19 bioclimatic variables from WorldClim across the six regions. The first component (PC1) accounted for the 32.8% variation in the data and was negatively associated with precipitation variables (precipitation seasonality, precipitation of the wettest month and wettest quarter and precipitation of the coldest quarter). The second component (PC2) accounted for 24.7% of the variation and was positively associated with temperature variables (annual mean temperature, max and mean temperature of the warmest month and warmest quarter and mean temperature of the wettest quarter). Bioclimatic variables are coded as follows: *bio1* = Annual Mean Temperature, *bio2* = Mean Diurnal Range (Mean of monthly (max temp - min temp)), *bio3* = Isothermality (BIO2/BIO7) (×100), *bio4* = Temperature Seasonality (standard deviation ×100), *bio5* = Max Temperature of Warmest Month, *bio6* = Min Temperature of Coldest Month, *bio7* = Temperature Annual Range (BIO5-BIO6), *bio8* = Mean Temperature of Wettest Quarter, *bio9* = Mean Temperature of Driest Quarter, *bio10* = Mean Temperature of Warmest Quarter, *bio11* = Mean Temperature of Coldest Quarter, *bio12* = Annual Precipitation, *bio13* = Precipitation of Wettest Month, *bio14* = Precipitation of Driest Month, *bio15* = Precipitation Seasonality (Coefficient of Variation), *bio16* = Precipitation of Wettest Quarter, *bio17* = Precipitation of Driest Quarter, *bio18* = Precipitation of Warmest Quarter, *bio19* = Precipitation of Coldest Quarter.

**
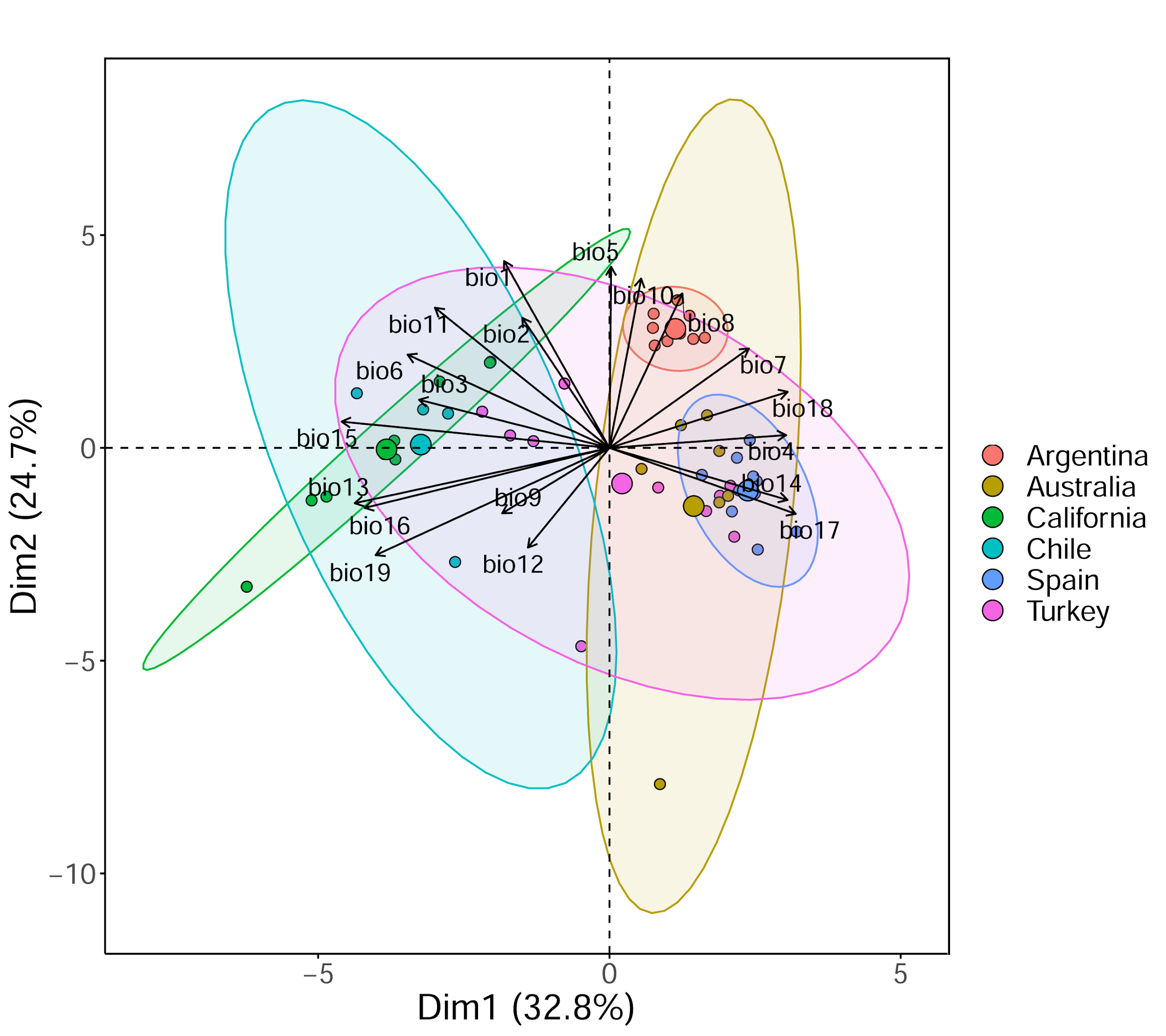
**

**Figure S3.** Radar plots showing the differentiation in climatic space among the six regions. Bioclimatic variables are coded as follows: *bio1* = Annual Mean Temperature, *bio2* = Mean Diurnal Range (Mean of monthly (max temp - min temp)), *bio3* = Isothermality (BIO2/BIO7) (×100), *bio4* = Temperature Seasonality (standard deviation ×100), *bio5* = Max Temperature of Warmest Month, *bio6* = Min Temperature of Coldest Month, *bio7* = Temperature Annual Range (BIO5-BIO6), *bio8* = Mean Temperature of Wettest Quarter, *bio9* = Mean Temperature of Driest Quarter, *bio10* = Mean Temperature of Warmest Quarter, *bio11* = Mean Temperature of Coldest Quarter, *bio12* = Annual Precipitation, *bio13* = Precipitation of Wettest Month, *bio14* = Precipitation of Driest Month, *bio15* = Precipitation Seasonality (Coefficient of Variation), *bio16* = Precipitation of Wettest Quarter, *bio17* = Precipitation of Driest Quarter, *bio18* = Precipitation of Warmest Quarter, *bio19* = Precipitation of Coldest Quarter. Turkey and Spain are the two native regions whereas Argentina, Chile, California and Australia are the introduced regions.


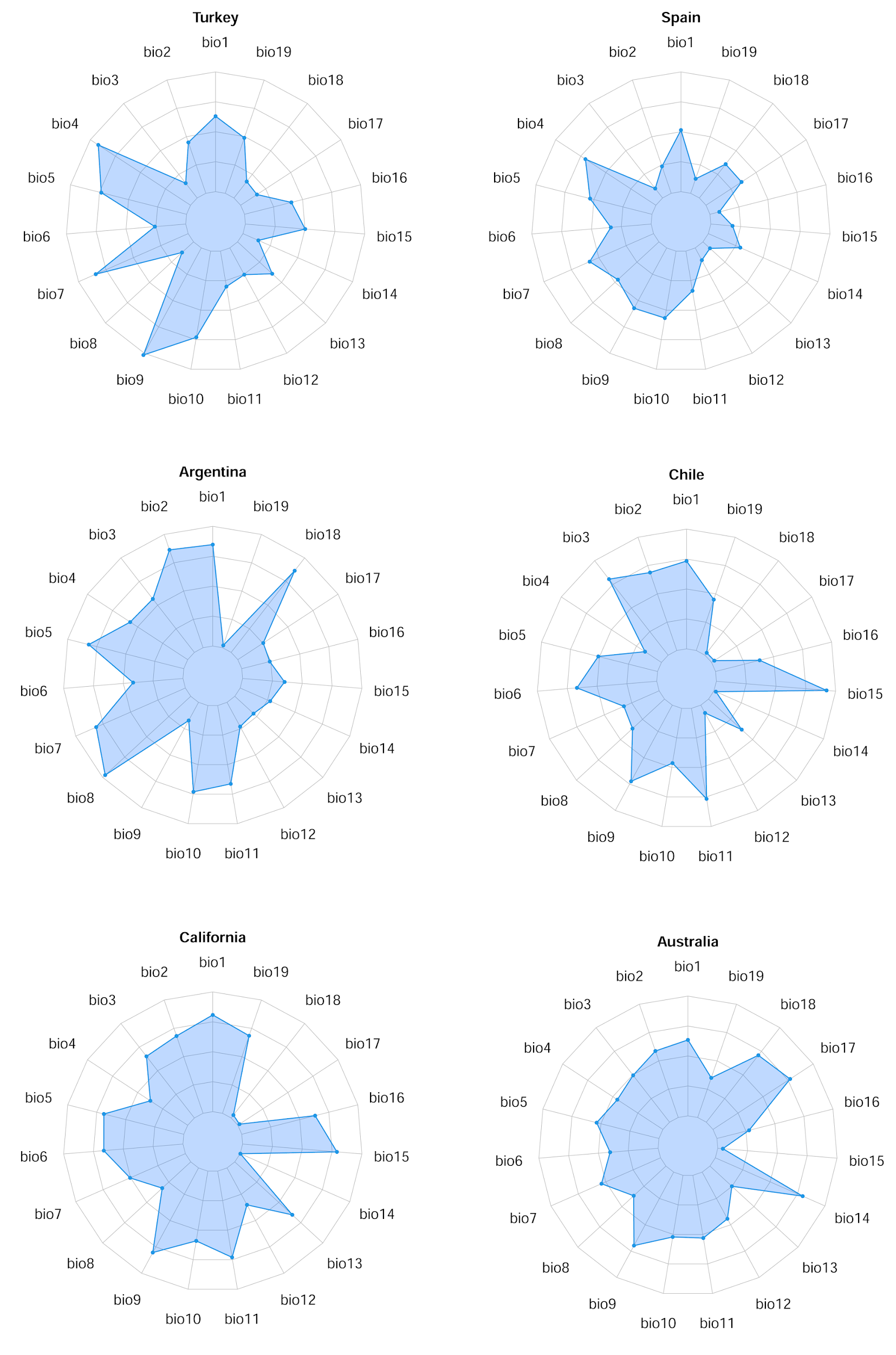


**Table S4.** Generalized linear mixed model summary output between phenotypic traits that showed significant differences between regions as presented in Table S3 (capitula number and seed mass) and bioclimatic variables (principal components). Germination time was used as a co-variable in the model to account for putative growth differences among individuals.

| **Spain** | | |  |
| --- | --- | --- | --- |
| *Predictors* | *Estimates* | *CI* | *p* |
| (Intercept) | 174.36 | 116.26 – 232.46 | **<0.001** |
| Germination | -18.93 | -33.56 – -4.31 | **0.011** |
| PC1 | -0.49 | -7.50 – 6.51 | 0.891 |
| PC2 | 1.92 | -9.26 – 13.10 | 0.736 |
| PC3 | -4.6 | -18.73 – 9.54 | 0.524 |

1. Capitula number

| **Turkey** | | |  |
| --- | --- | --- | --- |
| *Predictors* | *Estimates* | *CI* | *p* |
| (Intercept) | 118.94 | 74.63 – 163.26 | **<0.001** |
| Germination | -3.13 | -14.06 – 7.81 | 0.577 |
| PC1 | -10.91 | -14.63 – -7.20 | **<0.001** |
| PC2 | 0.01 | -5.46 – 5.47 | 0.998 |
|  |  |  |  |

| **Argentina** | | |  |
| --- | --- | --- | --- |
| *Predictors* | *Estimates* | *CI* | *p* |
| (Intercept) | 60.99 | 29.00 – 92.99 | **<0.001** |
| Germination | 0.83 | -6.65 – 8.31 | 0.828 |
| PC1 | -0.07 | -4.52 – 4.37 | 0.974 |
| PC2 | -2.89 | -8.42 – 2.65 | 0.306 |
| PC3 | -3.23 | -12.50 – 6.03 | 0.494 |

| **Chile** | | |  |
| --- | --- | --- | --- |
| *Predictors* | *Estimates* | *CI* | *p* |
| (Intercept) | 106.4 | 58.38 – 154.42 | **<0.001** |
| Germination | -10.17 | -20.77 – 0.44 | 0.073 |
| PC1 | 6.23 | 3.46 – 8.99 | **<0.001** |
| PC2 | 1.84 | -2.35 – 6.03 | 0.398 |

| **Australia** | | |  |
| --- | --- | --- | --- |
| *Predictors* | *Estimates* | *CI* | *p* |
| (Intercept) | 63.23 | 9.24 – 117.21 | **0.022** |
| Germination | 2.48 | -9.45 – 14.41 | 0.684 |
| PC1 | 2.22 | -1.41 – 5.86 | 0.231 |
| PC2 | -0.85 | -8.78 – 7.08 | 0.833 |

| **California** | | |  |
| --- | --- | --- | --- |
| *Predictors* | *Estimates* | *CI* | *p* |
| (Intercept) | 102.25 | 57.39 – 147.12 | **<0.001** |
| Germination | -5.35 | -17.83 – 7.14 | 0.405 |
| PC1 | -0.36 | -3.34 – 2.63 | 0.816 |
| PC2 | 8.8 | 4.17 – 13.43 | **<0.001** |
| PC3 | -1.97 | -7.78 – 3.83 | 0.508 |

| **Spain** | | |  |
| --- | --- | --- | --- |
| *Predictors* | *Estimates* | *CI* | *p* |
| (Intercept) | 1.58 | 0.91 – 2.26 | **<0.001** |
| Germination | -0.01 | -0.21 – 0.18 | 0.891 |
| PC1 | -0.03 | -0.08 – 0.03 | 0.371 |
| PC2 | -0.01 | -0.11 – 0.09 | 0.887 |
| PC3 | -0.08 | -0.20 – 0.04 | 0.206 |

1. Seed mass

| **Turkey** | | |  |
| --- | --- | --- | --- |
| *Predictors* | *Estimates* | *CI* | *p* |
| (Intercept) | 2.2 | 1.76 – 2.63 | **<0.001** |
| Germination | -0.19 | -0.30 – -0.08 | **0.001** |
| PC1 | 0.08 | 0.04 – 0.12 | **<0.001** |
| PC2 | -0.08 | -0.17 – 0.01 | 0.093 |

| **Argentina** | | |  |
| --- | --- | --- | --- |
| *Predictors* | *Estimates* | *CI* | *p* |
| (Intercept) | 2.07 | 1.62 – 2.52 | **<0.001** |
| Germination | -0.03 | -0.13 – 0.08 | 0.648 |
| PC1 | 0.01 | -0.05 – 0.06 | 0.744 |
| PC2 | -0.07 | -0.14 – -0.00 | **0.037** |
| PC3 | -0.02 | -0.14 – 0.10 | 0.728 |

|  | **Chile** | | |
| --- | --- | --- | --- |
| *Predictors* | *Estimates* | *CI* | *p* |
| (Intercept) | 0.69 | -0.34 – 1.71 | 0.22 |
| Germination | 0.22 | -0.01 – 0.46 | 0.09 |
| PC1 | -0.02 | -0.09 – 0.04 | 0.448 |
| PC2 | 0.04 | -0.07 – 0.16 | 0.473 |

| **California** | | |  |
| --- | --- | --- | --- |
| *Predictors* | *Estimates* | *CI* | *p* |
| (Intercept) | 1.83 | 1.16 – 2.51 | **<0.001** |
| Germination | 0.13 | -0.06 – 0.32 | 0.191 |
| PC1 | 0 | -0.04 – 0.04 | 0.989 |
| PC2 | -0.06 | -0.11 – -0.01 | **0.018** |
| PC3 | 0.01 | -0.06 – 0.09 | 0.777 |

| **Australia** | | |  |
| --- | --- | --- | --- |
| *Predictors* | *Estimates* | *CI* | *p* |
| (Intercept) | 2.24 | 1.58 – 2.90 | **<0.001** |
| Germination | -0.09 | -0.25 – 0.08 | 0.311 |
| PC1 | -0.04 | -0.07 – -0.00 | 0.052 |
| PC2 | 0.03 | -0.03 – 0.10 | 0.352 |

**Table S5**. Component loadings of 19 bioclimatic variables based on principal component analysis of WorldClim data across the six regions. Bioclimatic variables are coded as follows: *bio1* = Annual Mean Temperature, *bio2* = Mean Diurnal Range (Mean of monthly (max temp - min temp)), *bio3* = Isothermality (BIO2/BIO7) (×100), *bio4* = Temperature Seasonality (standard deviation ×100), *bio5* = Max Temperature of Warmest Month, *bio6* = Min Temperature of Coldest Month, *bio7* = Temperature Annual Range (BIO5-BIO6), *bio8* = Mean Temperature of Wettest Quarter, *bio9* = Mean Temperature of Driest Quarter, *bio10* = Mean Temperature of Warmest Quarter, *bio11* = Mean Temperature of Coldest Quarter, *bio12* = Annual Precipitation, *bio13* = Precipitation of Wettest Month, *bio14* = Precipitation of Driest Month, *bio15* = Precipitation Seasonality (Coefficient of Variation), *bio16* = Precipitation of Wettest Quarter, *bio17* = Precipitation of Driest Quarter, *bio18* = Precipitation of Warmest Quarter, *bio19* = Precipitation of Coldest Quarter. Variables with high loadings (>0.25 and <-0.25) on the principal components are bolded.

| **Region** | **PC** | **Variation** | **bio1** | **bio2** | **bio3** | **bio4** | **bio5** | **bio6** | **bio7** | **bio8** | **bio9** | **bio10** | **bio11** | **bio12** | **bio13** | **bio14** | **bio15** | **bio16** | **bio17** | **bio18** | **bio19** |
| --- | --- | --- | --- | --- | --- | --- | --- | --- | --- | --- | --- | --- | --- | --- | --- | --- | --- | --- | --- | --- | --- |
| TR | PC1 | 57% | -0.28 | -0.0 | -0.18 | 0.20 | -0.24 | **-0.29** | 0.14 | **-0.29** | -0.27 | -0.27 | **-0.29** | -0.07 | -0.15 | 0.27 | -0.24 | -0.13 | 0.26 | **0.28** | -0.13 |
|  | PC2 | 28% | -0.12 | -0.30 | -0.11 | -0.20 | -0.21 | -0.03 | -0.29 | -0.07 | -0.15 | -0.15 | -0.07 | **0.40** | **0.36** | 0.09 | 0.22 | **0.36** | 0.12 | 0.05 | **0.36** |
| SP | PC1 | 55% | 0.14 | -0.29 | -0.24 | -0.17 | -0.21 | 0.22 | -0.26 | 0.24 | -0.28 | 0.03 | 0.18 | 0.23 | 0.27 | 0.29 | -0.22 | 0.22 | 0.29 | 0.29 | -0.08 |
|  | PC2 | 24% | 0.40 | 0.00 | 0.00 | 0.03 | 0.22 | 0.29 | 0.01 | 0.25 | -0.16 | 0.37 | 0.38 | -0.22 | -0.12 | -0.12 | 0.25 | -0.21 | -0.11 | -0.10 | -0.37 |
|  | PC3 | 14% | 0.15 | 0.15 | -0.25 | 0.49 | 0.35 | -0.09 | 0.30 | 0.08 | 0.10 | 0.36 | -0.01 | 0.26 | 0.22 | 0.03 | 0.01 | 0.28 | 0.09 | 0.07 | 0.28 |
| AR | PC1 | 52% | 0.27 | -0.21 | 0.05 | -0.23 | 0.00 | 0.29 | -0.20 | 0.24 | **0.30** | 0.26 | **0.30** | 0.29 | **0.30** | 0.20 | 0.09 | 0.25 | 0.16 | 0.25 | 0.16 |
|  | PC2 | 30% | 0.20 | 0.30 | -0.08 | 0.25 | **0.38** | -0.15 | 0.30 | 0.17 | 0.10 | 0.20 | 0.10 | 0.07 | 0.08 | -0.24 | **0.36** | 0.18 | **-0.31** | 0.20 | **-0.31** |
|  | PC3 | 10% | 0.14 | 0.00 | **-0.54** | 0.22 | 0.26 | 0.00 | 0.14 | **0.34** | 0.11 | 0.19 | 0.11 | -0.16 | -0.07 | 0.18 | -0.29 | -0.27 | 0.21 | -0.26 | 0.21 |
| CHI | PC1 | 73% | **-0.26** | 0.24 | **0.26** | -0.19 | **-0.26** | -0.25 | 0.10 | -0.26 | **-0.26** | **-0.26** | -0.26 | 0.18 | 0.01 | 0.25 | -0.26 | 0.06 | **0.26** | 0.26 | 0.11 |
|  | PC2 | 26% | 0.03 | -0.18 | -0.02 | -0.30 | -0.05 | 0.11 | **-0.40** | 0.08 | 0.02 | 0.00 | 0.08 | 0.31 | **0.44** | -0.14 | 0.09 | **0.43** | 0.02 | 0.08 | **0.39** |
| CA | PC1 | 54% | **-0.31** | 0.11 | **0.30** | -0.29 | -0.28 | -0.04 | -0.26 | -0.05 | **-0.31** | **-0.31** | 0.11 | 0.26 | 0.28 | 0.00 | 0.24 | 0.27 | -0.01 | 0.13 | 0.27 |
|  | PC2 | 27% | -0.05 | 0.29 | -0.14 | 0.20 | 0.21 | **-0.40** | 0.25 | **-0.42** | 0.13 | 0.12 | **-0.41** | 0.22 | 0.21 | 0.00 | 0.03 | 0.21 | 0.04 | 0.16 | 0.21 |
|  | PC3 | 14% | 0.09 | **0.33** | 0.09 | -0.04 | -0.01 | -0.25 | 0.02 | -0.09 | -0.02 | -0.03 | 0.05 | -0.17 | -0.07 | 0.00 | **0.40** | -0.09 | **-0.59** | **-0.49** | -0.09 |
| AU | PC1 | 72% | -0.27 | -0.20 | -0.21 | -0.16 | -0.26 | -0.22 | -0.18 | -0.26 | -0.21 | -0.26 | -0.26 | 0.26 | 0.26 | 0.16 | 0.19 | 0.26 | 0.20 | 0.21 | 0.26 |
|  | PC2 | 21% | -0.02 | 0.32 | 0.21 | 0.36 | 0.09 | -0.28 | 0.35 | 0.13 | -0.18 | 0.05 | -0.09 | 0.09 | 0.05 | 0.39 | -0.32 | 0.04 | 0.32 | 0.29 | 0.01 |

**Table S6.** Outlier SNPs between the native and non-native ranges identified by BayeScan, OutFLANK and PCAdapt. Locus naming system: digits before the low dash represent the tag number, digits after the low dash represent the position of SNP on the tag. SNPs identified by more than one method are bolded. Sequence information are available in NCBI under BioProject ID: PRJNA950038.

| **Method** | **Total Loci** | **Locus name** |
| --- | --- | --- |
| BayeScan OutFLANK PCAdapt | 3 | **27727_100, 1225_114, 29308_43** |
| BayeScan OutFLANK | 3 | **10004_58, 16569_113, 22138_114** |
| BayeScan PCAdapt | 1 | **4600_75** |
| OutFLANK PCAdapt | 12 | **12536_93, 22138_85, 4126_107, 801_101, 22566_60, 29948_96, 9430_93, 7770_46, 7770_73, 11870_114, 21765_16, 23976_118** |
| BayeScan | 4 | 737_45, 2498_84, 20745_61, 12279_26 |
| OutFLANK | 18 | 8691_20, 1911_54, 22973_109, 18176_10, 1911_94, 5441_75, 2719_83, 17802_114, 3854_32, 11958_91, 7306_115, 28262_8, 20213_10, 24865_33, 27727_14, 11958_56, 5441_108,1911_117 |
| PCAdapt | 122 | 24602_55, 46333_98, 22040_78, 770_10, 5906_37, 30939_83, 80_49, 3370_129, 25633_94, 26165_52, 925_39, 12147_112, 9744_90, 6768_68, 24577_80, 23302_66, 24833_108, 3276_66, 14843_20, 11156_91, 4600_96, 437_32, 17425_51, 25212_66, 22138_40, 3370_26, 1477_85, 3370_66, 11870_82, 3162_16, 516_117, 5527_45, 2128_46, 11240_47, 21609_40, 45761_59, 18473_10, 11870_88, 31478_28, 24339_44, 14280_107, 11495_74, 28398_45, 2669_72, 26271_111, 21567_8, 28110_85, 1917_72, 617949_57, 12289_23, 3790_34, 1065_80, 9708_54, 16866_37, 28320_58, 8464_76, 24142_70, 28110_69, 18788_42, 7421_8, 26276_25, 11821_121, 22176_78, 6595_91, 12536_81, 1335_96, 6370_22, 743_75, 17970_106, 26881_24, 39255_47, 16686_18, 6027_57, 192_96, 3855_114, 12059_86, 25618_7, 12536_42, 28589_110, 27027_83, 27147_49, 24009_74, 883207_47, 8464_112, 925_21, 29502_92, 12841_30, 13492_85, 879292_10, 38172_26, 25159_39, 22388_112, 26229_68, 5487_99, 1335_113, 408_95, 3642_31, 3973_37, 17970_96, 7374_6, 23469_96, 17863_76, 13086_10, 3642_13, 21077_98, 21077_22, 23302_30, 39255_12, 24731_61, 2004_35, 2004_90, 20907_98, 10084_49, 12535_63, 28631_28, 1024_74, 12466_64, 28592_74, 11471_87, 29902_92, 31330_89, 17147_7 |

**Figure S4.** Venn diagram of outlier loci identified by BayeScan, OutFLANK and PCAdapt

*
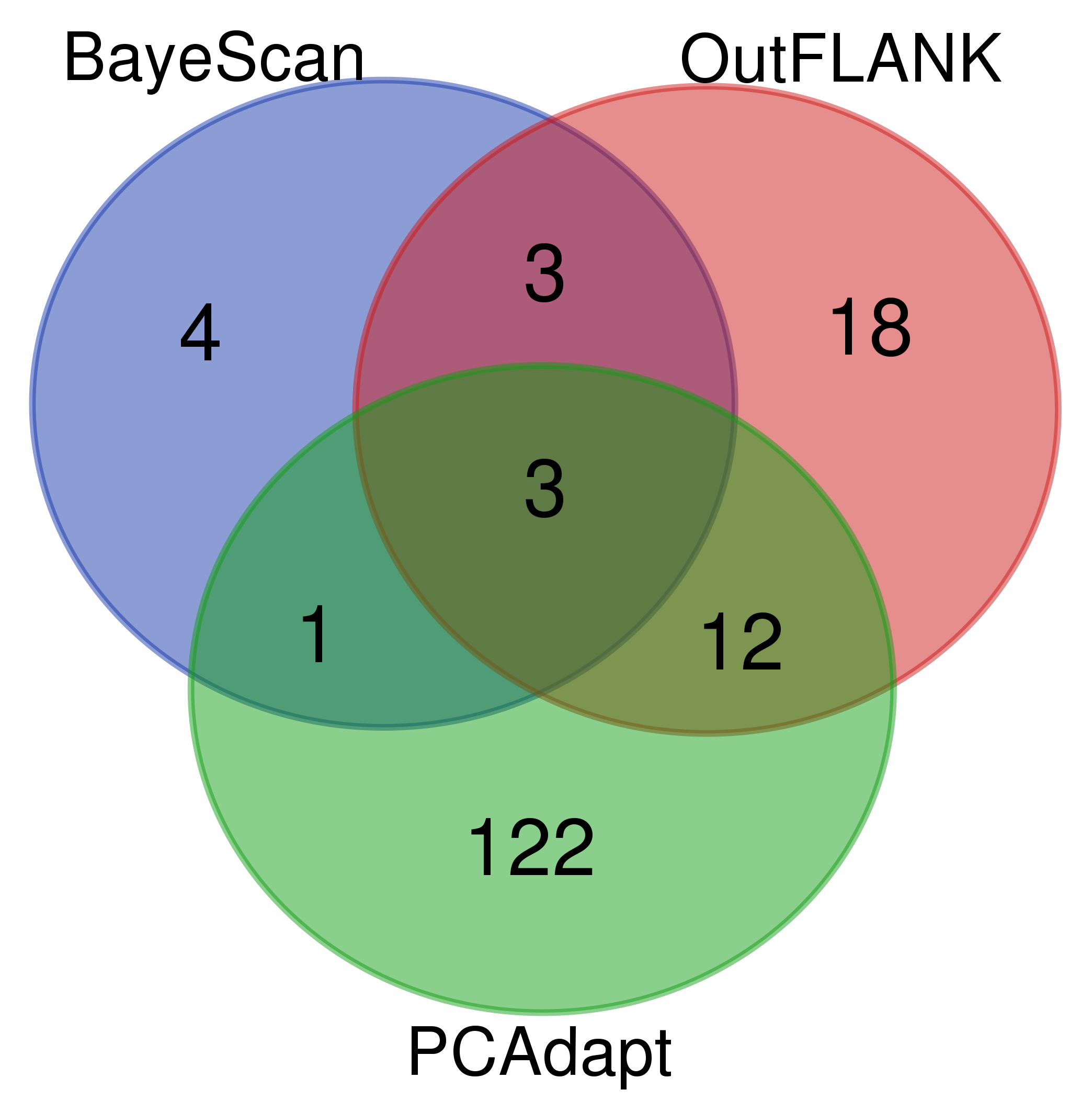
*

**Table S7.** Evanno method using delta K (rate of change in likelihood among models) to differentiate among number of populations inferred from neutral structure analysis. K value demonstrating peak in delta K is bolded.

| **K** | **Reps** | **Mean LnP(K)** | **Stdev LnP(K)** | **Ln'(K)** | **\|Ln''(K)\|** | **ΔK** |
| --- | --- | --- | --- | --- | --- | --- |
| 1 | 10 | -181358.960000 | 2.341509 | — | — | — |
| **2** | **10** | **-176524.470000** | **1.449176** | **4834.490000** | **2672.830000** | **1844.379137** |
| 3 | 10 | -174362.810000 | 8.395693 | 2161.660000 | 242.860000 | 28.926735 |
| 4 | 10 | -172444.010000 | 10.642833 | 1918.800000 | 542.550000 | 50.977969 |
| 5 | 10 | -171067.760000 | 11.445737 | 1376.250000 | 508.520000 | 44.428770 |
| 6 | 10 | -170200.030000 | 127.892733 | 867.730000 | 145.810000 | 1.140096 |
| 7 | 10 | -169478.110000 | 877.618196 | 721.920000 | 288.620000 | 0.328867 |
| 8 | 10 | -168467.570000 | 33.725725 | 1010.540000 | — | — |

**Fig S5.** STRUCTURE plots for all tested K’s (1-8): a) major models, b) minor models.

**
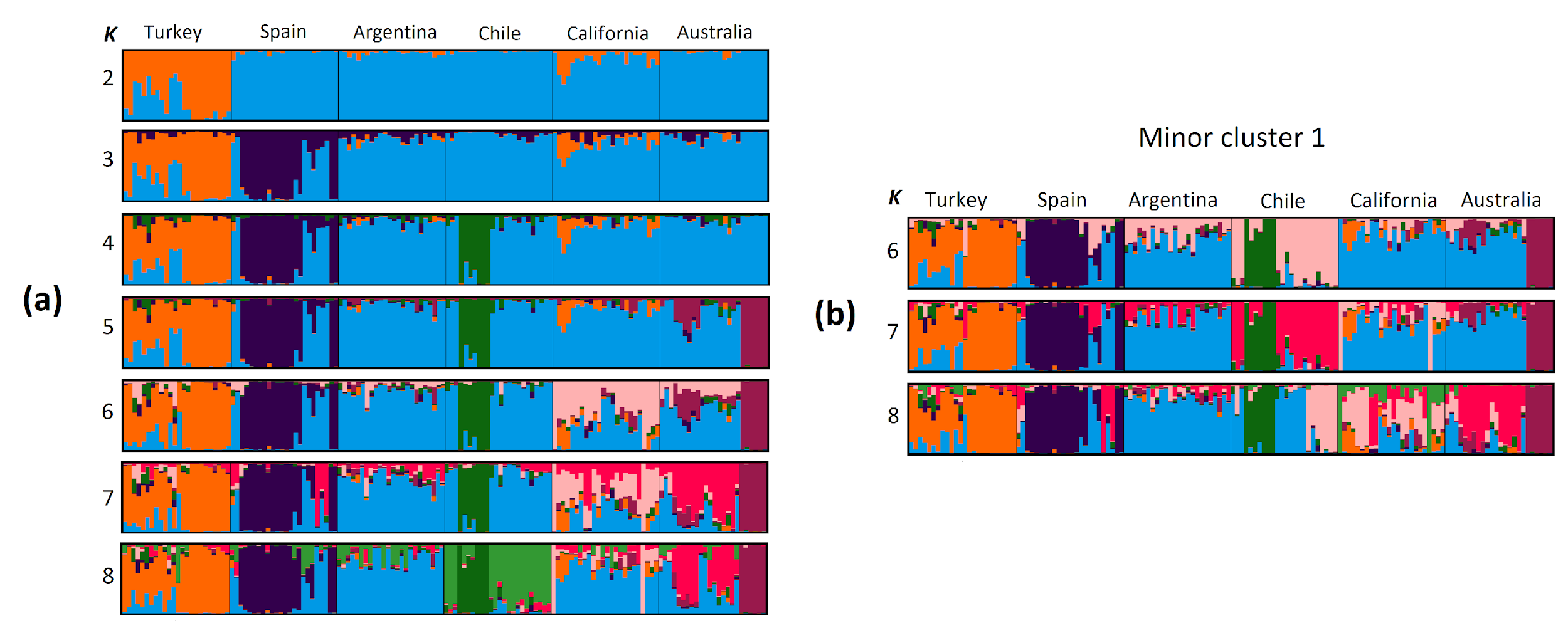
**

**Table S8**. *P*_ST_-*F*_ST_ pair-wise region comparisons of phenotypic and neutral genetic differentiation at seven morphological traits in *C*. *solstitialis*. The significance of the differences for each comparison are indicated by the Bayesian credibility interval and highlighted in bold. Regions are abbreviated by two-letters country code or three letters country code (i.e. Chile).

| **Trait** | **comparison** | **mean P_ST_ (97.5% CI)** | **mean F_ST_ (95% CI)** | **P_ST_ - F_ST_ Bayesian credibility interval**  **(97.5% CI)** |
| --- | --- | --- | --- | --- |
| Days to bolting | TR *vs* SP | 0.03 (0.00 – 0.11) | 0.08 (0.06 – 0.11) | -0.05 (-0.08 – 0.02) |
| Days to bolting | TR *vs* AR | 0.00 (0.00 – 0.05) | 0.07 (0.05 – 0.10) | -0.07 (-0.07 – (-0.02) |
| Days to bolting | TR *vs* CHI | 0.00 (0.00 – 0.02) | 0.08 (0.06 – 0.11) | -0.08 (-0.08 – (-0.05) |
| Days to bolting | TR *vs* CA | 0.00 (0.00 – 0.01) | 0.06 (0.04 – 0.08) | -0.06 (-0.06 – (-0.04) |
| Days to bolting | TR *vs* AU | 0.01 (0.00 – 0.07) | 0.08 (0.06 – 0.11) | -0.07 (-0.08 – (-0.01) |
| Days to bolting | SP *vs* AR | 0.00 (0.00 – 0.01) | 0.02 (0.01 – 0.04) | -0.02 (-0.02 – (-0.01) |
| Days to bolting | SP *vs* CHI | 0.04 (0.00 – 0.17) | 0.04 (0.02 – 0.06) | 0.00 (-0.04 – 0.13) |
| Days to bolting | SP *vs* CA | 0.01 (0.00 – 0.07) | 0.03 (0.02 – 0.05) | -0.02 (-0.03 – 0.03) |
| Days to bolting | SP *vs* AU | 0.00 (0.00 – 0.01) | 0.03 (0.02 – 0.05) | -0.03 (-0.03 – (-0.01) |
| Days to bolting | AR *vs* CHI | 0.01 (0.00 – 0.07) | 0.02 (0.01 – 0.04) | -0.01 (-0.02 – 0.04) |
| Days to bolting | AR *vs* CA | 0.01 (0.00 – 0.07) | 0.02 (0.01 – 0.03) | -0.00 (-0.02 – 0.05) |
| Days to bolting | AR *vs* AU | 0.00 (0.00 – 0.01) | 0.02 (0.00 – 0.03) | -0.01 (-0.02 – (-0.00) |
| Days to bolting | CHI *vs* CA | 0.00 (0.00 – 0.02) | 0.03 (0.02 – 0.05) | -0.03 (-0.03 – (-0.00) |
| Days to bolting | CHI *vs* AU | 0.01 (0.00 – 0.10) | 0.03 (0.02 –0.05) | -0.01 (-0.33 – 0.07) |
| Days to bolting | CA *vs* AU | 0.02 (0.00 – 0.10) | 0.02 (0.01– 0.04) | -0.00 (-0.02 – 0.08) |
| Days to first flower | TR *vs* SP | 0.02 (0.00 – 0.09) | 0.08 (0.06 – 0.11) | -0.06 (-0.08 – 0.00) |
| Days to first flower | TR *vs* AR | 0.00 (0.00 – 0.01) | 0.07 (0.05 – 0.10) | -0.07 (-0.07 – (-0.06) |
| Days to first flower | TR *vs* CHI | 0.01 (0.00 – 0.08) | 0.08 (0.06 – 0.11) | -0.07 (-0.08 – (-0.00) |
| Days to first flower | TR *vs* CA | 0.00 (0.00 – 0.05) | 0.06 (0.04 – 0.08) | -0.05 (-0.06 – (-0.00) |
| Days to first flower | TR *vs* AU | 0.06 (0.00 – 0.17) | 0.08 (0.06 – 0.11) | -0.02 (-0.08 – 0.08) |
| Days to first flower | SP *vs* AR | 0.02 (0.00 – 0.10) | 0.02 (0.01 – 0.04) | -0.00 (-0.02 – 0.07) |
| Days to first flower | SP *vs* CHI | 0.03 (0.00 – 0.16) | 0.04 (0.02 – 0.06) | -0.00 (-0.04 – 0.12) |
| Days to first flower | SP *vs* CA | 0.07 (0.00 – 0.18) | 0.03 (0.02 – 0.05) | 0.04 (-0.03 – 0.15) |
| Days to first flower | SP *vs* AU | 0.00 (0.00 – 0.01) | 0.03 (0.02 – 0.05) | -0.03 (-0.03 – (-0.01) |
| Days to first flower | AR *vs* CHI | 0.00 (0.00 – 0.06) | 0.02 (0.01 – 0.04) | -0.01 (-0.02 – 0.04) |
| Days to first flower | AR *vs* CA | 0.00 (0.00 – 0.04) | 0.02 (0.01 – 0.03) | -0.01 (-0.02 – 0.02) |
| Days to first flower | AR *vs* AU | 0.07 (0.00 – 0.18) | 0.02 (0.00 – 0.03) | 0.05 (-0.02 – 0.16) |
| Days to first flower | CHI *vs* CA | 0.00 (0.00 – 0.03) | 0.03 (0.02 – 0.05) | -0.03 (-0.03 – (-0.00) |
| Days to first flower | CHI *vs* AU | 0.11 (0.00 – 0.27) | 0.03 (0.02 – 0.05) | 0.07 (-0.03 – 0.24) |
| Days to first flower | CA *vs* AU | 0.13 (0.01 – 0.26) | 0.02 (0.01 – 0.04) | 0.10 (-0.00 – 0.23) |
| Final plant height | TR *vs* SP | 0.00 (0.00 – 0.02) | 0.08 (0.06 – 0.11) | -0.08 (-0.08 – (-0.06) |
| Final plant height | TR *vs* AR | 0.00 (0.00 – 0.02) | 0.07 (0.05 – 0.10) | -0.07 (-0.07 – (-0.05) |
| Final plant height | TR *vs* CHI | 0.08 (0.00 – 0.21) | 0.08 (0.06 – 0.11) | -0.00 (-0.08 – 0.12) |
| Final plant height | TR *vs* CA | 0.01 (0.00 – 0.06) | 0.06 (0.04 – 0.08) | -0.05 (-0.06 – (-0.00) |
| Final plant height | TR *vs* AU | 0.00 (0.00 – 0.02) | 0.08 (0.06 – 0.11) | -0.08 (-0.08 – (-0.06) |
| Final plant height | SP *vs* AR | 0.00 (0.00 – 0.03) | 0.02 (0.01 – 0.04) | -0.02 (-0.02 – 0.00) |
| Final plant height | SP *vs* CHI | 0.11 (0.00 – 0.24) | 0.04 (0.02 – 0.06) | 0.06 (-0.04 – 0.20) |
| Final plant height | SP *vs* CA | 0.01 (0.00 – 0.08) | 0.03 (0.02 – 0.05) | -0.01 (-0.03 – 0.04) |
| Final plant height | SP *vs* AU | 0.00 (0.00 – 0.03) | 0.03 (0.02 – 0.05) | -0.03 (-0.03 – (-0.00) |
| Final plant height | AR *vs* CHI | 0.08 (0.00 – 0.21) | 0.02 (0.01 – 0.04) | 0.05 (-0.02 – 0.19) |
| Final plant height | AR *vs* CA | 0.01 (0.00 – 0.07) | 0.02 (0.01 – 0.03) | -0.00 (-0.02 – 0.04) |
| Final plant height | AR *vs* AU | 0.00 (0.00 – 0.02) | 0.02 (0.00 – 0.03) | -0.01 (-0.02 – 0.00) |
| Final plant height | CHI *vs* CA | 0.02 (0.00 – 0.13) | 0.03 (0.02 – 0.05) | -0.00 (-0.03 – 0.09) |
| Final plant height | CHI *vs* AU | 0.1 (0.00 – 0.24) | 0.03 (0.02 – 0.05) | 0.06 (-0.03 – 0.21) |
| Final plant height | CA *vs* AU | 0.00 (0.00 – 0.05) | 0.02 (0.01 – 0.04) | -0.01 (-0.02 – 0.03) |
| Capitula | TR *vs* SP | 0.00 (0.00 – 0.02) | 0.08 (0.06 – 0.11) | -0.08 (-0.08 – (-0.05) |
| Capitula | TR *vs* AR | 0.07 (0.00 – 0.16) | 0.07 (0.05 – 0.10) | -0.00 (-0.07 – 0.08) |
| Capitula | TR *vs* CHI | 0.09 (0.00 – 0.23) | 0.08 (0.06 – 0.11) | 0.00 (-0.08– 0.14) |
| Capitula | TR *vs* CA | 0.01 (0.00 – 0.06) | 0.06 (0.04 – 0.08) | -0.05 (-0.06 – (-0.00) |
| Capitula | TR *vs* AU | 0.05 (0.00 – 0.15) | 0.08 (0.06 – 0.11) | -0.03 (-0.08 – 0.06) |
| Capitula | SP *vs* AR | 0.04 (0.00 – 0.13) | 0.02 (0.01 – 0.04) | 0.02 (-0.02 – 0.10) |
| Capitula | SP *vs* CHI | 0.06 (0.00 – 0.19) | 0.04 (0.02 – 0.06) | 0.02 (-0.04 – 0.15) |
| Capitula | SP *vs* CA | 0.00 (0.00 – 0.03) | 0.03 (0.02 – 0.05) | -0.02 (-0.03 – 0.00) |
| Capitula | SP *vs* AU | 0.03 (0.00 – 0.12) | 0.03 (0.02 – 0.05) | -0.00 (-0.03 – 0.08) |
| Capitula | AR *vs* CHI | 0.00 (0.00 – 0.04) | 0.02 (0.01 – 0.04) | -0.01 (-0.02 – 0.02) |
| Capitula | AR *vs* CA | 0.03 (0.00 – 0.12) | 0.02 (0.01 – 0.03) | 0.01 (-0.02 – 0.10) |
| Capitula | AR *vs* AU | 0.00 (0.00 – 0.02) | 0.02 (0.00 – 0.03) | -0.01 (-0.02 – 0.00) |
| Capitula | CHI *vs* CA | 0.07 (0.00 – 0.21) | 0.03 (0.02 – 0.05) | 0.04 (-0.03 – 0.18) |
| Capitula | CHI *vs* AU | 0.00 (0.00 – 0.05) | 0.03 (0.02 – 0.05) | -0.02 (-0.03 – 0.01) |
| Capitula | CA *vs* AU | 0.02 (0.00 – 0.11) | 0.02 (0.01 – 0.04) | 0.00 (-0.02 – 0.08) |
| Largest spine | TR *vs* SP | 0.06 (0.00 – 0.15) | 0.08 (0.06 – 0.11) | -0.02 (-0.08 – 0.06) |
| Largest spine | TR *vs* AR | 0.01 (0.00 – 0.06) | 0.07 (0.05 – 0.10) | -0.06 (-0.07 – (-0.00) |
| Largest spine | TR *vs* CHI | 0.00 (0.00 – 0.04) | 0.08 (0.06 – 0.11) | -0.08 (-0.08 – (-0.04) |
| Largest spine | TR *vs* CA | 0.00 (0.00 – 0.02) | 0.06 (0.04 – 0.08) | -0.05 (-0.06 – (-0.03) |
| Largest spine | TR *vs* AU | 0.11 (0.02 – 0.23) | 0.08 (0.06 – 0.11) | 0.02 (-0.06 – 0.14) |
| Largest spine | SP *vs* AR | 0.01 (0.00 – 0.07) | 0.02 (0.01 – 0.04) | -0.01 (-0.02 – 0.04) |
| Largest spine | SP *vs* CHI | 0.07 (0.00 – 0.21) | 0.04 (0.02 – 0.06) | 0.03 (-0.04 – 0.17) |
| Largest spine | SP *vs* CA | 0.06 (0.00 – 0.15) | 0.03 (0.02 – 0.05) | 0.02 (-0.03 – 0.12) |
| Largest spine | SP *vs* AU | 0.01 (0.00 – 0.07) | 0.03 (0.02 – 0.05) | -0.02 (-0.03 – 0.03) |
| Largest spine | AR *vs* CHI | 0.02 (0.00 – 0.11) | 0.02 (0.01 – 0.04) | 0.00 (-0.02 – 0.09) |
| Largest spine | AR *vs* CA | 0.01 (0.00 – 0.07) | 0.02 (0.01 – 0.03) | -0.01 (-0.02 – 0.05) |
| Largest spine | AR *vs* AU | 0.05 (0.01 – 0.15) | 0.02 (0.00 – 0.03) | 0.03 (-0.01– 0.13) |
| Largest spine | CHI *vs* CA | 0.00 (0.00 – 0.04) | 0.03 (0.02 – 0.05) | -0.02 (-0.03 – 0.01) |
| Largest spine | CHI *vs* AU | 0.11 (0.00 – 0.27) | 0.03 (0.02 – 0.05) | 0.08 (-0.02 – 0.24) |
| Largest spine | CA *vs* AU | 0.10 (0.01 – 0.23) | 0.02 (0.01 – 0.04) | 0.08 (-0.00 – 0.20) |
| Seed mass | TR *vs* SP | 0.02 (0.00 – 0.10) | 0.08 (0.06 – 0.11) | -0.06 (-0.08 – 0.01) |
| **Seed mass** | **TR *vs* AR** | **0.20 (0.08 – 0.31)** | **0.07 (0.05 – 0.10)** | **0.12 (0.01 – 0.24)** |
| Seed mass | TR *vs* CHI | 0.02 (0.00 – 0.13) | 0.08 (0.06 – 0.11) | -0.06 (-0.08 – 0.04) |
| **Seed mass** | **TR *vs* CA** | **0.45 (0.32 – 0.57)** | **0.06 (0.04 – 0.08)** | **0.39 (0.26 – 0.50)** |
| Seed mass | TR *vs* AU | 0.18 (0.03 – 0.34) | 0.08 (0.06 – 0.11) | 0.09 (-0.05 – 0.26) |
| Seed mass | SP *vs* AR | 0.11 (0.02 – 0.23) | 0.02 (0.01 – 0.04) | 0.08 (-0.00 – 0.20) |
| Seed mass | SP *vs* CHI | 0.01 (0.00 – 0.08) | 0.04 (0.02 – 0.06) | -0.02 (-0.04 – 0.04) |
| **Seed mass** | **SP *vs* CA** | **0.40 (0.25 – 0.53)** | **0.03 (0.02 – 0.05)** | **0.36 (0.22 – 0.50)** |
| Seed mass | SP *vs* AU | 0.11 (0.00 – 0.27) | 0.03 (0.02 – 0.05) | 0.07 (-0.03 – 0.23) |
| Seed mass | AR *vs* CHI | 0.06 (0.00 – 0.22) | 0.02 (0.01 –0.04) | 0.03 (-0.02 – 0.20) |
| Seed mass | AR *vs* CA | 0.08 (0.01 – 0.19) | 0.02 (0.01 – 0.03) | 0.06 (-0.01 – 0.17) |
| Seed mass | AR *vs* AU | 0.01 (0.00 – 0.06) | 0.02 (0.00 – 0.03) | -0.00 (-0.02 – 0.04) |
| **Seed mass** | **CHI *vs* CA** | **0.32 (0.11 – 0.52)** | **0.03 (0.02 – 0.05)** | **0.29 (0.07 – 0.48)** |
| Seed mass | CHI *vs* AU | 0.06 (0.00 – 0.25) | 0.03 (0.02 – 0.05) | 0.03 (-0.03 – 0.21) |
| **Seed mass** | **CA *vs* AU** | **0.17 (0.02 – 0.34)** | **0.02 (0.01 – 0.04)** | **0.14 (0.003– 0.31)** |
